# MOCOVIDOA: a novel multi-objective coronavirus disease optimization algorithm for solving multi-objective optimization problems

**DOI:** 10.1007/s00521-023-08587-w

**Published:** 2023-05-02

**Authors:** Asmaa M. Khalid, Hanaa M. Hamza, Seyedali Mirjalili, Khaid M. Hosny

**Affiliations:** 1 Department of Information Technology, Faculty of Computers and Informatics, Zagazig University, Zagazig, 44519 Egypt; 2 Centre for Artificial Intelligence Research and Optimisation, Torrens University Australia, Fortitude Valley, Brisbane, QLD 4006 Australia

**Keywords:** Coronavirus, Multi-objective, Frameshifting, Dominance, Convergence, Coverage

## Abstract

A novel multi-objective Coronavirus disease optimization algorithm (MOCOVIDOA) is presented to solve global optimization problems with up to three objective functions. This algorithm used an archive to store non-dominated POSs during the optimization process. Then, a roulette wheel selection mechanism selects the effective archived solutions by simulating the frameshifting technique Coronavirus particles use for replication. We evaluated the efficiency by solving twenty-seven multi-objective (21 benchmarks & 6 real-world engineering design) problems, where the results are compared against five common multi-objective metaheuristics. The comparison uses six evaluation metrics, including IGD, GD, MS, SP, HV, and delta *p* ($\Delta \mathrm{P}$). The obtained results and the Wilcoxon rank-sum test show the superiority of this novel algorithm over the existing algorithms and reveal its applicability in solving multi-objective problems.

## Introduction

Nature has provided humanity with methodologies and approaches for solving highly complex problems [44]. The GA is a well-known nature-inspired algorithm in the optimization field [19], where scientists mimic the evolution of natural biological organisms to find high-quality solutions to optimize problems [20]. The amazing success of the GA in solving various optimization problems motivated researchers to develop additional heuristics such as ACO [14], DE [43], CSA [23], and PSO [24]. Such algorithms usually search for the best solution by generating all solutions to the problem, evaluating them, and then updating them until a maximum number of iterations is reached. Despite the efficiency of these algorithms, they face many difficulties when solving some real problems, such as multiple objectives [33, 34], many objectives [22], constraints [3], uncertainty [7], and local optima [27], which requires a modification of these metaheuristics to handle such difficulties.

Optimization techniques are divided into single- and multi-objective [10]. The first type achieves the optimal solution by comparing the utilized objective function. In the case of multi-objective optimization, the goal is to find the POSs [36, 37]. Solving multi-objective problems is classified into two main groups: Priori and Posteriori [17]. In the a priori method, sufficient information must be provided before making any decision. This information can aggregate all the objectives into a single one by defining the single-objective function as a weighted sum of the normalized costs associated with each objective [35]. In such a case, a single-objective algorithm can be suitable, as it is straightforward and does not require any modification of the basic algorithm. However, it has disadvantages, such as consulting with the decision-maker to determine the preferred weights. A posteriori method aims to find a set of POSs. Then, apply the decision-making [40]. It can find all the POSs in one run. This approach maintains the formulation of the multi-objective problem and does not need to aggregate the different objectives or determine a set of weights. NSGA [12], MOPSO [8], MOEA/D [50], PAES [28], MOSMA [21], MOJS [6], MOMPA [18], MOVPA [39], and MOSOA [13] are examples of the well-regarded multi-objective optimization algorithms. These algorithms suffer from limitations such as slow convergence and inefficiency in solving complex higher-dimensional problems or when the number of objectives increases. Interested readers are referred to [36–38], and [30] for more algorithms.

COVIDOA is one of the most recently proposed swarm intelligence metaheuristics [25, 26]. It is inspired by Coronavirus particles' behavior inside the human body. A coronavirus particle goes through a replication life cycle within a human host cell, creating more copies of itself that can eventually infect and damage more healthy human cells. The key stages of the Coronavirus replication lifecycle include virus attachment to human cells through spike protein [16], virus entry and uncoating [31], virus replication through the ribosomal frameshifting technique [4], virus mutation [5], and new virion assembly and release [32]. COVIDOA has been employed in various optimization problems and has shown superior performance [25, 25, 26, 26] due to its excellent exploration and exploitation capabilities and high convergence speed.

This work presents a novel multi-objective COIVDOA (MOCOVIDOA) which generalizes the original COVIDOA for solving optimization problems with up to three objective functions. The proposed algorithm utilizes the archive concept to store and retrieve the POSs. The roulette wheel selection selects a non-dominated solution from the archive. The reasons for developing the multi-objective version of COVIDOA are as follows:

- Engineering design problems inherently have multiple conflicting objectives with multiple constraints. Single-objective techniques are usually failed to optimize all objectives.
- The NFL theorem [13] demonstrates that no single optimization method can find the optimum solution for all optimization problems. This theory motivated us to develop new approaches that may perform better in solving complex optimization problems.
- The outstanding performance of COVIDOA and BCOVIDOA in single-objective optimization [25, 25, 26, 26] motivated the authors to derive the MOCOVIDOA to solve multi-objective real-world problems.
- Most of the current approaches are suitable only for unconstrained problems. The COVIDOA is proposed to solve both constrained and unconstrained problems.

This paper is organized as follows: An overview of multi-objective optimization and a concise description of the single-objective COVIDOA are presented in Sect. 2. The proposed MOCOVIDOA is introduced in Sect. 3. We discussed the benchmark and engineering test problems and the obtained results in Sect. 4. Finally, the conclusion is presented in Sect. 5.

## Preliminaries

### Multi-objective optimization

Multi-objective optimization involves optimizing a mathematical problem with multiple objective functions, where optimizing these functions simultaneously is the main challenge. A multi-objective optimization can be represented as a minimization optimization problem [33, 34]:

1 $$Minimize: F\left(\overrightarrow{t}\right)=\{{f}_{1}\left(t\right),{ f}_{2}\left(t\right), ..., {f}_{o}\left(t\right)\}$$

2 $$Subject~ to:{ g}_{i}\left(t\right)\ge 0, 1\le i\le p$$

3 $${h}_{i}\left(t\right)=0, 1\le i\le q$$

4 $${L}_{i}\le {t}_{i}\le {U}_{i}, 1\le i\le D$$

where *o* is the number of objective functions, *p* is the number of inequality constraints, *q* is the number of equality constraints, *D* is the problem dimension, ${g}_{i}$ is the *i*th inequality constraint, ${h}_{i}$ is the *i*th equality constraint, and ${L}_{i}$ and ${U}_{i}$ are the lower and upper bounds of the *i*th variable*.*

It should be noted that, in single-objective optimization, it is easy to find the optimal solution to the problem because of a single objective. In contrast, multi-objective optimization requires finding solutions representing the best trade-off among the different objectives [11].

To compare solutions for multiple objectives, relational operators are not suitable due to the existence of multiple conflicting objectives; instead, the dominance operator [49] is utilized as follows:

In a minimization problem, a solution *x*_*1*_ is said to dominate another solution *x*_*2*_ (denoted as${x}_{1}\prec {x}_{2}$) if and only if:

5 $$\forall i \in \left\{1, \dots , o\right\}, {f}_{i}\left({x}_{1}\right)\le {f}_{i}\left({x}_{2}\right), and$$

6 $$\exists i\in \left\{1, \dots , o\right\}, {f}_{i}\left({x}_{1}\right)<{f}_{i}\left({x}_{2}\right).$$

For the maximization problem, a solution *x*_*1*_ dominates *x*_*2*_ if:

7 $$\forall i \in \left\{1, \dots , o\right\}, {f}_{i}\left({x}_{1}\right)\ge {f}_{i}\left({x}_{2}\right)\quad{and}$$

8 $$\exists i\in \left\{1, \dots , o\right\}, {f}_{i}\left({x}_{1}\right)>{f}_{i}\left({x}_{2}\right).$$

A solution is better than another if it has the same fitness value as the other solution in all objectives and better fitness in at least one objective. A solution is called Pareto optimal if there is no other solution that dominates it as follows:

9 $$\nexists \overrightarrow{y}\in X\left|\overrightarrow{y} \right.\prec \overrightarrow{x}$$

**Fig. 1:**
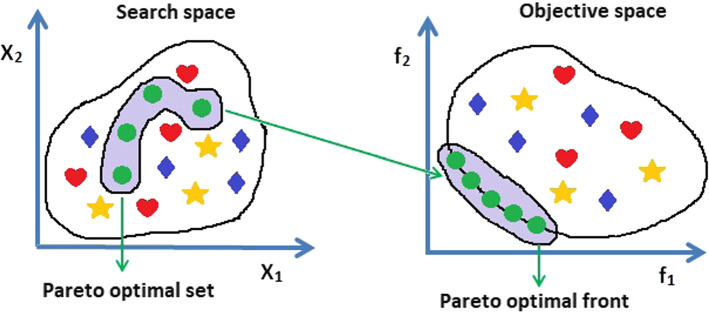
Search space and Objective space in multi-objective optimization

Each multi-objective function has a set of non-dominated solutions called the Pareto optimal set, defined as follows (see Fig. 1):

10 $${P}_{s}:=\{\overrightarrow{x}, \overrightarrow{y} \in X\left|\nexists \overrightarrow{y}\right.\prec \overrightarrow{x}\}$$

The set of Objective functions values for the Pareto optimal set is called the Pareto front as follows:

11 $${P}_{f}:=\{ f\left(\overrightarrow{x}\right)\left|\overrightarrow{x}\right. \in {P}_{s}\}$$

The general steps of almost all multi-objective optimization algorithms in the evolutionary computation field are similar. They begin the optimization process with an initial random population of solutions. Then they compare these solutions using the dominance operator to find the set of POSs and keep them in an archive. In the next iterations, each algorithm tries to improve the quality of the archive solutions to get the closest possible approximation to the true Pareto set [29].

There are two main conflicting objectives of all posterior multi-objective algorithms; convergence and coverage. Convergence refers to improving the quality of the non-dominated solutions obtained during the optimization process to improve their accuracy compared to the true POSs. Coverage means improving the distribution of the non-dominated solutions to cover the entire Pareto optimal front. Focusing on only one of the two objectives will negatively affect the other, so an effective algorithm must balance these two criteria well [49].

### Coronavirus disease optimization algorithm

**Fig. 2:**
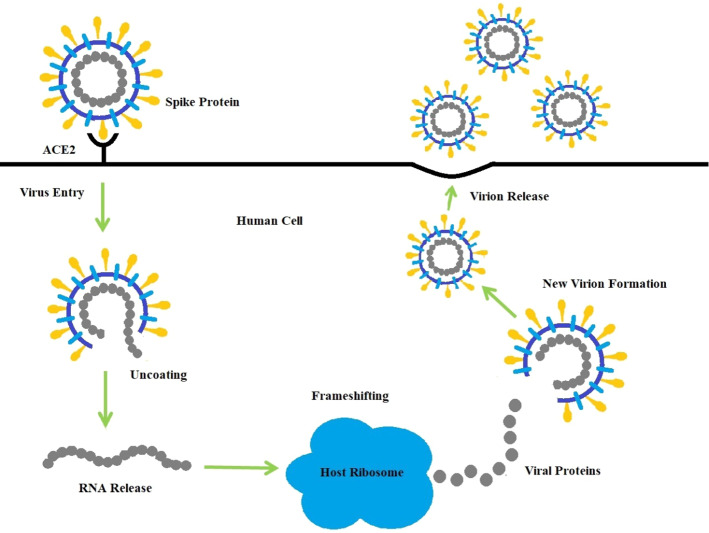
The replication lifecycle of Coronavirus

COVIDOA is a recent evolutionary metaheuristic inspired by the replication mechanism of Coronavirus when hijacking human body cells [25, 26]. The replication process of Coronavirus is divided into four main stages as follows (see Fig. 2 as well):

#### Virus entry and uncoating

When a human is infected with COVID, the Coronavirus particles bind to the human cell via one of its structural proteins, spike protein. After getting inside the human cell, the virus contents are released.

#### Virus replication

The virus aims to make more copies to hijack as many as possible human healthy cells. The virus uses what is referred to as the ribosomal frameshifting technique for replication [4]. Frameshifting moves the reading frame of a virus protein sequence to another reading frame, leading to the creation of many different protein sequences. The newly generated viral proteins are then merged to form new virus particles. There are many types of frameshifting techniques; however, the most popular is + 1 frameshifting as follows [45]:

-  + 1 frameshifting technique

The elements of the parent virus particle (parent solution) are moved in the right direction in 1 step. As a result of + 1 frameshifting, the first element is lost. In the proposed algorithm, the first element is set to a random value in the range [Lb, Ub] as follows:

12 $$S_k (1)=\mathrm{rand}(\mathrm{Lb},\mathrm{Ub}),$$

13 $$S_k (2:\mathrm{D})=\mathrm{ P}(1:\mathrm{D}-1),$$

where Lb and Ub are the lower and upper bounds for the variables in each solution,* P* is the parent sequence, and ${\mathrm{S}}_{\mathrm{k}}$ is the generated viral protein number* k*.

#### Virus mutation

Coronavirus uses the mutation technique to resist the human immune system. In the COVID algorithm, the mutation is applied to the previously created virus particle (solution) to produce a new one as follows:

14 $$ Zi = \left\{ {\begin{array}{*{20}l}    r \hfill & {if\ {\text{ }}r\ and\ \left( {0,1} \right)< MR} \hfill  \\    {X_{i} } \hfill & {otherwise} \hfill  \\   \end{array} } \right. $$

MR refers to the mutation rate. Where* X* is the solution before mutation, *Z* is the mutated solution,* X*i and* Z*i are the ith element in the old and new solutions, respectively, *i* = 1, …, D*,* where *D* is the problem dimension,* r* is a random value in the range [Lb, Ub]. Coronavirus has a very low mutation rate (1 × 10–6), as mentioned in [2], however, using such a low mutation rate will limit the algorithm's ability to explore the search space. Thus, the mutation rate in the proposed algorithm is set to a larger value in the range [0.005, 0.5], which helps explore new promising regions of the search space.

#### New virion release

The newly created virus particle leaves the infected cell targeting new healthy cells. In the proposed algorithm, if the fitness of the new solution is better than the parent solution fitness, the parent solution is replaced by the new one; otherwise, the parent solution remains as follows:

15 $${P}_{i}^{t}=\left\{\begin{array}{l}{Z}_{i}^{t}\quad if f({Z}_{i}^{t})f({P}_{i}^{t})\\ {P}_{i}^{t}\quad otherwise\end{array}\right.$$

where ${P}_{i}^{t}$ is the *i*th parent (parent particle) in the *t*th iteration, and ${Z}_{i}^{t}$ is the *i*th generated child (new virion) in the *t*th iteration.

**Fig. 3:**
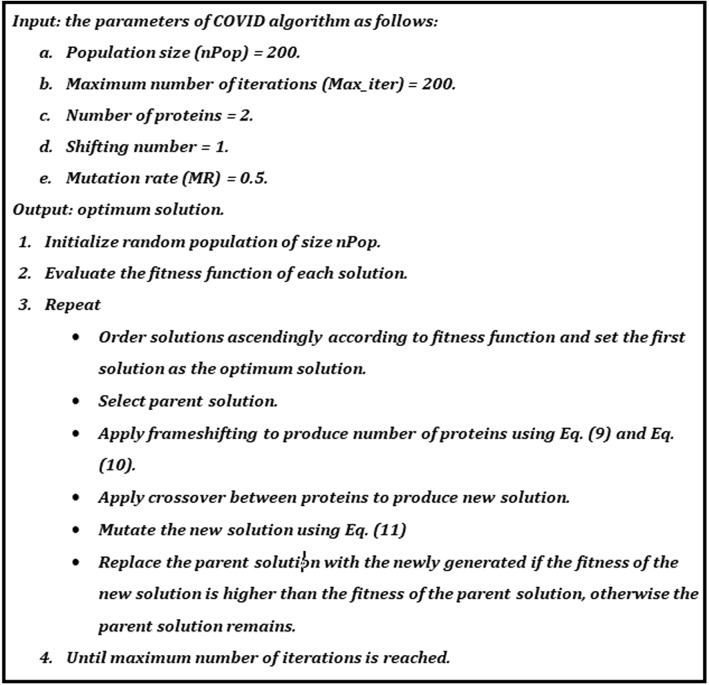
The pseudo-code of the COVIDOA algorithm

The pseudocode of the COVID algorithm is shown in Fig. 3.

As an evolutionary algorithm, COVIDOA has many advantages, such as:

1. Its concept is easy to understand.
2. It searches from a population of points, not a single point.
3. It can handle complex problems and parallelism.
4. It is robust to the local minima/maxima problem.

Despite these advantages, COVIDOA suffers from limitations such as its high time complexity compared with other algorithms due to mutation, crossover, and frameshifting processes. Important parameters such as mutation rate, crossover probability, and frameshifting number will make it difficult for the algorithm to converge if they are not chosen appropriately.

## Multi-objective coronavirus optimization algorithm (MOCOVIDOA)

The search mechanism of MOCOVIDOA is the same as in COVIDOA, which is based on the replication mechanism of Coronavirus. MOCOVIDOA uses the dominance operator to compare solutions considering multiple criteria (objective functions). All the POSs obtained so far are stored in an archive. A controller is used to determine which solution should be kept in the archive and which should be removed, which is done similarly to MOPSO [8]. The archive controller works according to the following rules:

- If the archive is empty, the current solution should be accepted.
- If another solution dominates the archive, the particular solution should be removed.
- The particular solution should be stored in the archive if the external population does not dominate a solution.
- If new elements dominate solutions in the archive, they should be removed.

Two approaches must be done to improve the coverage of solutions in the Pareto optimal front; solutions from the least crowded regions in the archive must be selected to contribute to the improvement of others, and solutions with many neighbors should be thrown away from the archive. A leader selection mechanism is utilized to perform these two approaches as follows:

MOVOIDOA selects a solution from the least populated regions in the archive using the roulette wheel selection. The probability of selecting a solution is defined as follows:

16 $${P}_{i}=\frac{c}{{N}_{i}}$$

where *c* is a constant greater than 1, and ${N}_{i}$ represents the number of solutions in the vicinity of the *i*th solution.

The undesired solutions are those with many neighbors. The undesired solutions should be removed when the archive becomes full. The probability of selecting a solution for removal from the archive is defined as follows:

17 $${P}_{i}=\frac{{N}_{i}}{c}$$

In MOCOVIDOA, Eq. (15) should be replaced by the following equation because of multiple objectives.

18 $${P}_{i}^{t}={Z}_{i}^{t}~ if~ f({Z}_{i}^{t})\prec f({P}_{i}^{t})$$

where ${P}_{i}^{t}$ is the *i*th parent (parent particle) in the *t*th iteration, and ${Z}_{i}^{t}$ is the *i*th generated child (new virion) in the *t*th iteration.

The rest of the operators used in MOCOVIDOA are the same as those used in COVIDOA.

The computational complexity of MOCOVIDOA is estimated as follows:

1. The MOCOVIDOA requires $O\left(m \times n \right)$ for calculating the fitness functions of search agents, where *m* represents the number of objective functions, and *n* is the population size.
2. The MOCOVIDOA requires $O\left(m \times {n}_{\mathrm{archive}}\right)$ time to update the archive where ${n}_{archive}$ refers to the number of non-dominated solutions in the archive.
3. Steps 1 and 2 are repeated until the maximum number of iterations ($Max\_iter$) is reached.

Hence the time complexity of MOCOVIDOA is $O\left(Max\_iter\times m \times n \times {n}_{archive}\right)$, which is more expensive than the complexity of the original single-objective COVIDOA ($O\left(\mathrm{Max}\_\mathrm{iter}\times m \times n \times {n}_{\mathrm{archive}}\right)$) due to an additional archive of solutions and multiple objective functions.

The computational complexity of the proposed MOCOVIDOA is the same as that of the well-known multi-objective algorithms: MOPSO, NSGA-II, SPEA2, and PEAS. However, it is better than the complexity of other algorithms such as NSGA and SPEA, *O(mn*^*3*^*).* According to the space complexity, MOCOVIDOA requires the same space as other algorithms, such as MOPSO, but it needs more space compared to some algorithms, such as NSGA-II, due to creating the archive.

**Fig. 4:**
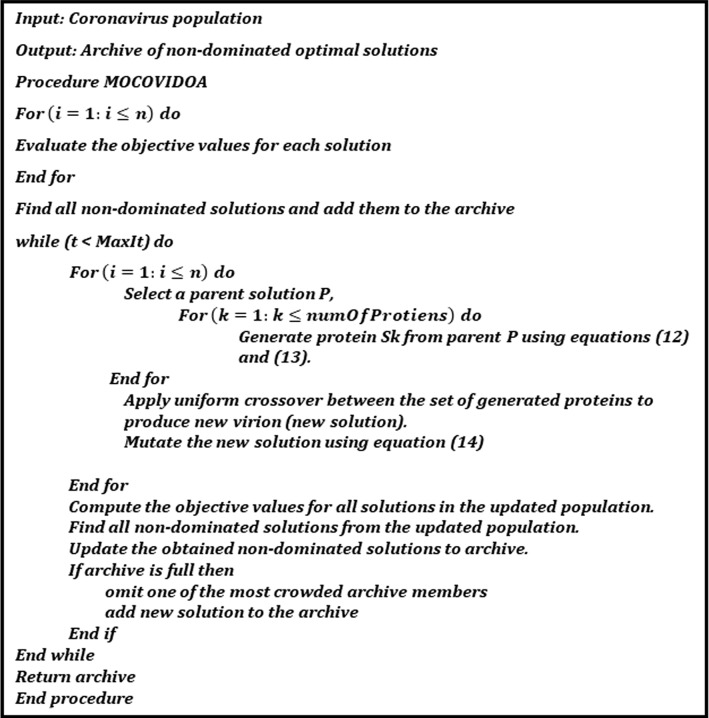
The pseudo-code of the MOCOVIDOA algorithm

**Fig. 5:**
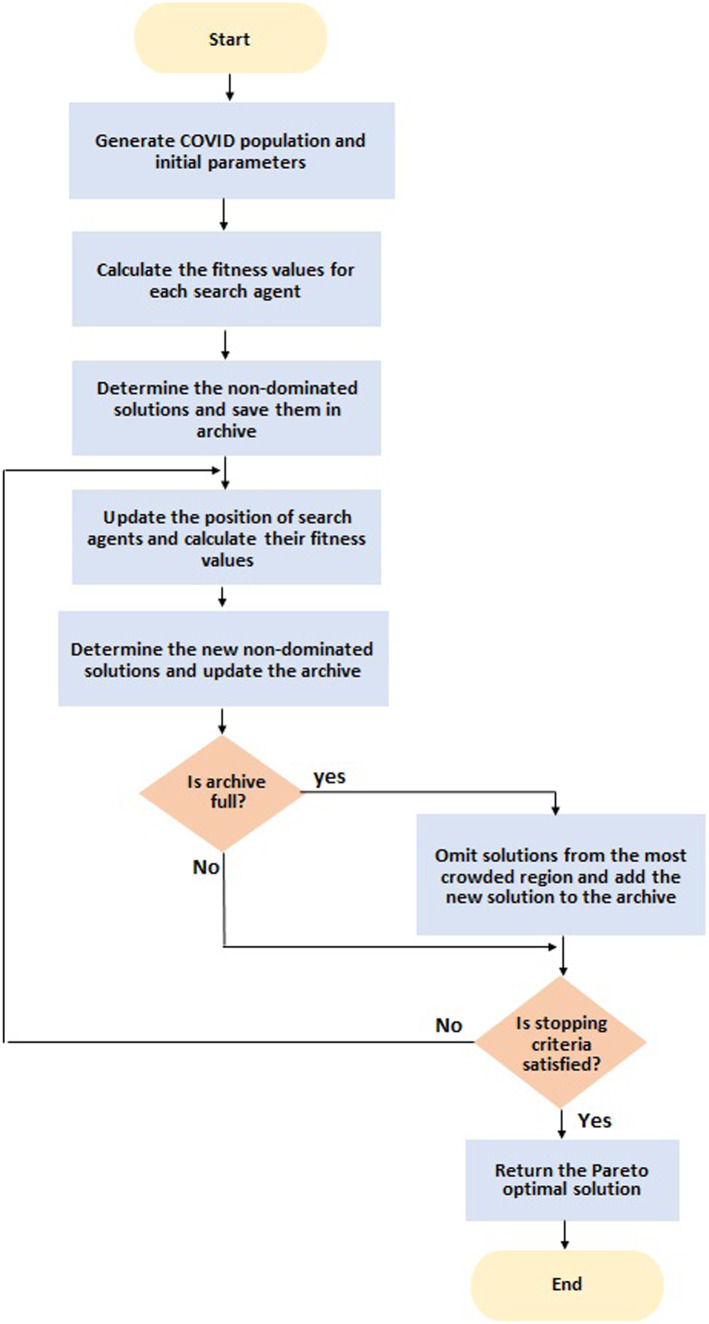
Flowchart of the proposed MOCOVIDOA

The pseudocode of the proposed MOCOVIDOA is presented in Fig. 4, and the flowchart is illustrated in Fig. 5.

## Experimental results and discussion

This section presents the performance of the proposed algorithm in solving 27 multi-objective problems with diverse characteristics and the comparisons with the state-of-the-art algorithms according to various evaluation metrics.

### Experimental setup

All the experiments were run on a laptop with the following specifications: Intel(R) Core(TM) i7-1065G7 processor, RAM of 8.0 GB size, and Windows 10 Ultimate 64-bit operating system. All the algorithms are developed using MATLAB R2016a development environment.

Twenty-seven benchmark case studies are used to evaluate the performance of MOCOVIDOA, as follows:

- Six unconstrained ZDT test suites, including ZDT1-ZDT4, ZDT6, and ZDT1, with linear Pareto front (Zitzler et al., 2002).
- Ten unconstrained CEC-2009 functions, including UF1-UF10 [51].
- Five constrained test functions include BNH, TNK, CONSTR, SRN, and OSY [47].
- Six real-world constrained engineering design problems include welded beam, speed reducer, four-bar truss, welded beam, and gear train design (Dept, 1991, [15, 42]. The details of these cases studied can be found in Appendices A, B, and C.

The reasons for selecting these benchmark problems for testing are as follows:

- The selected test functions have diverse characteristics, such as different Pareto optimal fronts (concave, convex, linear, and separated).
- Solving constrained real-world problems is more challenging.

The results are compared to five well-regarded and recent multi-objective approaches, including the multi-objective slime mold algorithm (MOSMA) [21], multi-objective ant lion optimizer (MOALO) [36, 37], multi-objective multi verse optimizer (MOMVO) [36, 37], multi-objective particle swarm optimization (MOPSO) [8], and multi-objective evolutionary algorithm based on decomposition (MOEA/D) [50] to verify the superiority of MOCOVIDOA. According to their original works, the parameters associated with these algorithms have remained the same.

All algorithms were run 30 times, and the best results are reported in the tables below. We have utilized 200 iterations and 100 search agents for all algorithms and a size of 100. The parameters of MOCOVIDOA are set as follows: mutation rate (MR) = 0.05, number of viral proteins ($\mathrm{num\, of\, Protiens}$) = 2, and the shifting number = 1. These parameter values yield the best results during the experiments.

### Evaluation metrics

Five metrics are utilized to evaluate the performance of MOCOVIDOA: *GD, IGD, Hypervolume,* and *Delta P* are used for measuring convergence of solutions, whereas *SP* and *MS* are used for measuring coverage as follows:

- Generational distance (GD):
  16 $$GD=\frac{\sqrt{\sum_{i=1}^{no}{d}_{i}^{2}}}{n}$$

Where *no* refers to the numerical solutions in the obtained Pareto optimal set, and *d*_*i*_ indicates the Euclidean distance between the *i*th obtained Pareto optimal solution and the closest true Pareto optimal solution in the reference set.

- Inverse generational distance (IGD):
  17 $$IGD=\frac{\sqrt{\sum_{i=1}^{nt}{({d}_{i}^{^{\prime}})}^{2}}}{n}$$

Where *nt* refers to the numerical solutions in the true Pareto optimal set, ${d}_{i}^{^{\prime}}$ Indicates the Euclidean distance between the *i*th true Pareto optimal solution and the closest Pareto optimal solution obtained in the reference set.

The GD and IGD metrics are used to quantify the convergence of solutions. In other words, they are used to measure to what extent the obtained POSs are close to the true POSs.

- Spacing (SP):
  18 $$SP\triangleq \sqrt{\frac{1}{no-1}\sum_{i=1}^{no}({\overline{d }- {d}_{i})}^{2}}$$

Where $\overline{d }$ is the average of all ${d}_{i}$. SP is used to measure the coverage of solutions where lower SP values indicate better coverage.

- Maximum spread (MS):
  19 $$MS=\sqrt{\sum_{i=1}^{o}\mathrm{max}(d({a}_{i} , {b}_{i}))}$$

The *o* refers to the number of s used to measure the coverage of the obtained POSs.

- Hyper volume (HV):
  20 $$HV=\mathrm{volume} (\bigcup_{i=1}^{A}{V}_{i})$$

HV represents the volume covered by the non-dominated solutions (A). Larger HV values indicate better convergence and diversity. The hypervolume ${\mathrm{V}}_{\mathrm{i}}$ of the *i*th solution is calculated for a reference point which can be calculated by creating a vector of the worst objective function values.

- Delta P ($$\Delta \mathrm{P}$$):
  21 $$\Delta P=max (mean \left(IGD\right), mean (GD))$$

### Results and discussion

#### Results of unconstrained test functions

The utilized unconstrained test functions comprise two well-known benchmark test suits, ZDT and CEC2009 (Zitzler et al., 2021, [5]. In the ZDT test suit, MOCOVIDOA is used to calculate the POSs for the 6 cases (ZDT1, ZDT2, ZDT3, ZDT4, ZDT6, and ZDT1 with linear Pareto front). The results of MOCOVIDOA are compared to five well-known approaches according to the previously mentioned evaluation metrics.

The results in Table 1 show the superiority of COVIDOA over its peers in most cases, and it achieves the best results in 30 of 36 test cases. However, in the remaining 6 cases, other algorithms slightly outperform it, such as MOSMA, MOALO, and MOMVO.

**Table 1:** Results for ZDT test suit

Problem	Metric	Algorithm					
		MOEA/D	MOMVO	MOPSO	MOALO	MOSMA	MOCOVIDOA
ZDT1	IGD	0.01269	0.01743	0.00511	0.07946	0.00453	**0.00340**
	GD	4.116e-04	8.021e-05	4.414e-04	1.662e-04	1.543e-04	**7.5687e-05**
	HV	0.71892	**0.70464**	0.71869	0.78804	0.71861	0.71851
	SP	0.011779	0.0079043	0.0090021	0.0065631	0.0050178	**0.0035578**
	MS	1.574	1.409	0.97889	1.286	1.6425	**0.67691**
	$\Delta P$	0.012697	0.018187	0.0051128	0.079464	0.0045356	**0.0034064**
ZDT2	IGD	0.0097974	0.018115	0.0053234	0.023649	0.0041723	**0.0037042**
	GD	0.0052154	1.236e-04	4.322e-04	3.536e-05	8.337e-05	**3.3945e-05**
	HV	0.43781	0.4256	0.44762	0.42727	0.44439	**0.40435**
	SP	0.0074974	0.0091459	0.0084532	0.0028212	0.0053305	**0.0020165**
	MS	1.7	1.3803	1.07878	1.0611	1.5497	**0.77377**
	$\Delta P$	0.0097974	0.018115	0.007453	0.096612	0.0041723	**0.0037042**
ZDT3	IGD	0.05256	0.0082337	0.0050806	0.024676	0.0048673	**0.0047739**
	GD	0.01557	5.185e-04	0.0031586	5.056e-04	5.052e-04	**3.1566e-04**
	HV	0.59914	0.6013	0.59962	0.61736	0.60079	**0.59946**
	SP	0.026697	0.010624	0.0578	0.012099	**0.0058912**	0.0078446
	MS	1.5095	0.96891	1.10654	1.5672	1.1718	0.88504
	$\Delta P$	0.07246	0.0082337	0.0072848	0.024676	0.0056132	**0.0042626**
ZDT4	IGD	0.092330	0.35701	0.0342531	0.42208	0.0128282	**0.011808**
	GD	0.00438	0.0090067	5.441e-04	0.016581	**6.176e-05**	8.6153e-05
	HV	0.71922	0.39349	0.72781	**0.30122**	0.72154	**0.70256**
	SP	0.045370	0.0039776	0.009278	0.0044905	0.0043051	**0.003771**
	MS	1.5673	0.97332	0.96757	0.99773	0.80244	**0.76189**
	$\Delta P$	0.15655	0.35701	0.082341	0.42208	**0.0088006**	0.01808
ZDT1LP	IGD	0.018910	0.0092952	0.004327	0.0271	0.004242	**0.0034987**
	GD	0.027091	3.118e-04	0.01381	2.894e-04	2.962e-04	**2.7715e-04**
	HV	0.58244	0.57328	0.58146	**0.55031**	0.58227	**0.58142**
	SP	0.008996	0.0084758	0.11332	0.012131	0.0051138	**0.0042528**
	MS	1.24644	1.1186	1.46054	1.5052	1.8384	**0.75907**
	$\Delta P$	0.056575	0.0092952	0.026368	0.02638	0.004242	**0.0036351**
ZDT6	IGD	0.008344	0.030016	0.0042184	0.012584	0.18633	**0.0039223**
	GD	0.003382	**5.34e-04**	0.003523	0.0011295	0.019226	**2.635e-05**
	HV	0.40342	0.36198	0.38769	0.37534	0.14189	0.38647
	SP	0.009760	0.020649	0.0077384	0.0075198	0.0070768	**0.0051128**
	MS	1.43560	0.83301	0.92123	1.6402	0.98581	0.96667
	$\Delta P$	0.007545	0.030016	0.010772	0.012584	0.22161	0.0038738

**Fig. 6:**
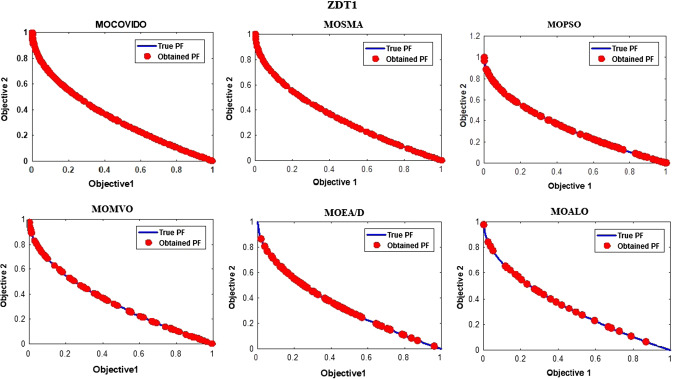
Obtained Pareto fronts for the ZDT1 test function

**Fig. 7:**
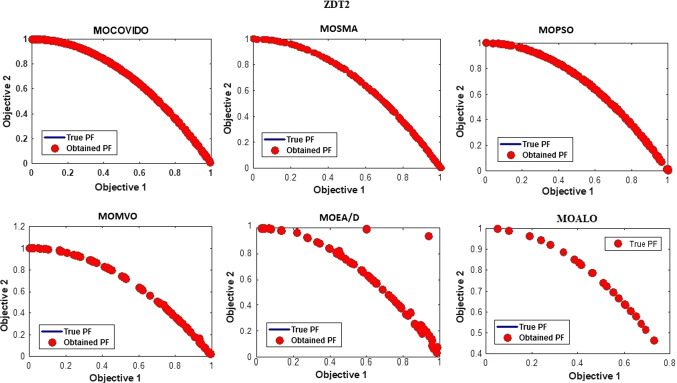
Obtained Pareto fronts for the ZDT2 test function

**Fig. 8:**
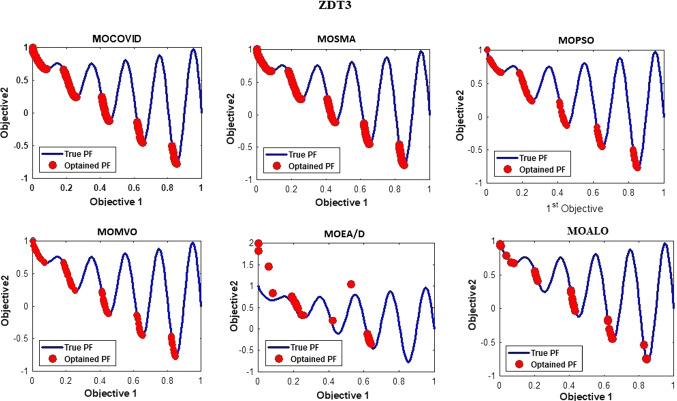
Obtained Pareto fronts for the ZDT3 test function

**Fig. 9:**
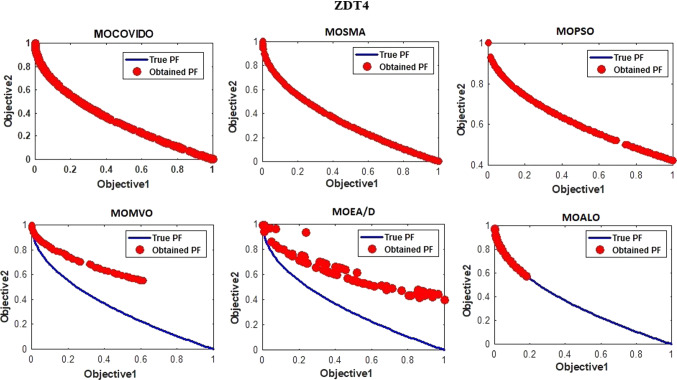
Obtained Pareto fronts for the ZDT4 test function

**Fig. 10:**
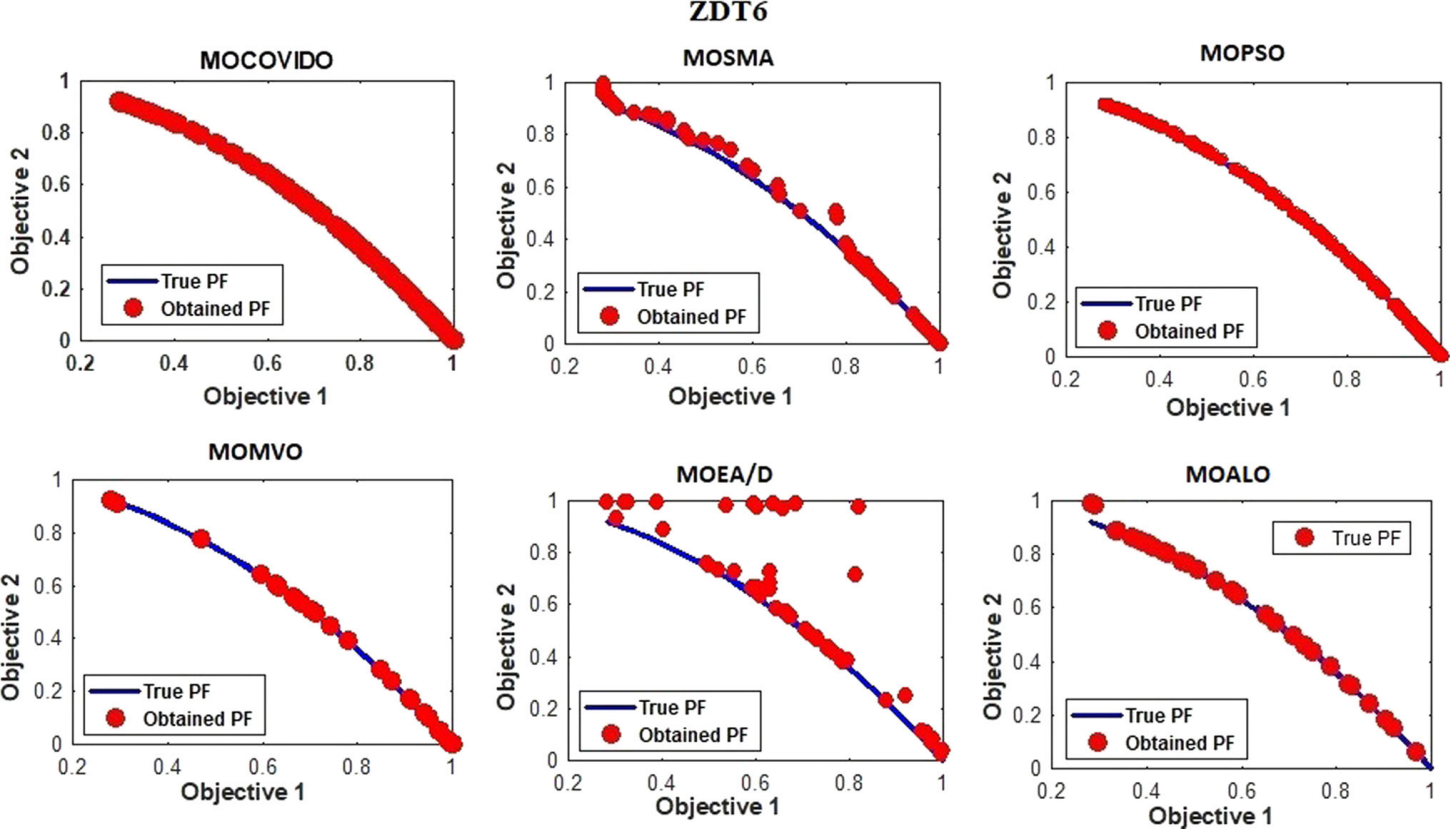
Obtained Pareto fronts for the ZDT6 test function

**Fig. 11:**
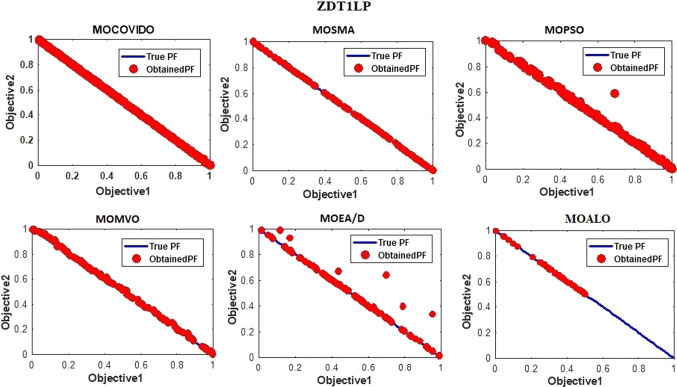
Obtained Pareto fronts for the ZDT1LP test function

The best Pareto fronts obtained from running all the ZDT test suit algorithms are shown in Figs. 6, 7, 8, 9, 10, 11. The figure shows high convergence and coverage of the Pareto optimum solutions obtained by the proposed algorithm since the obtained Pareto front is almost identical to the True Pareto front for all cases. The Pareto fronts of MOSMA and MOPSO are the closest to MOCOVIDOA, while MOMVO, MOALO, and MOEA/D show the lowest convergence and coverage. The comparison clearly shows that the Pareto front of MOCOVIDOA is better than other algorithms in all cases.

Additionally, we conducted the Wilcoxon rank sum test to prove the superiority of the proposed algorithm. The* p* values obtained from comparing MOCOVIDOA against other algorithms are shown in Table 2.

**Table 2:** The *P* values obtained from the rank-sum test on ZDT1-ZDT6

Problem	MOCOVIDOA vs MOEA/D	MOCOVIDOA vs MOMVO	MOCOVIDOA vs MOPSO	MOCOVIDOA vs MOALO	MOCOVIDOA vs MOSMA
ZDT1	0.0044	0.0025	0.0307	0.0085	0.0856
ZDT2	1.3017e-05	4.5512e–05	0.0106	7.3928e–04	0.0188
ZDT3	1.534e-09	2.0133e–08	0.00556	7.8416e–07	0.00169
ZDT4	5.638e-07	0.01482	0.0109	0.000282	1.5286e–05
ZDT1LP	2.3271e-05	0.03780	0.0085	2.5294e–07	0.01604
ZDT6	6.288e-07	4.301e–04	0.0052	2.533e–04	5.1408e–05

Smaller *p* values are strong evidence against the null hypothesis [46]. The null hypothesis is a type of hypothesis used in statistics that assumes no significant difference between the two methods' average values. All the p values shown in the table are less than 0.05 except the value obtained from comparing the performance of MOCOVIDOA with MOSMA for the ZDT1 test function, which shows that MOCOVIDOA is superior to MOSMA, MOPSO, MOMVO, MOALO, MOEA/D algorithms but has no significant difference with MOSMA for ZDT1 test function.

**Fig. 12:**
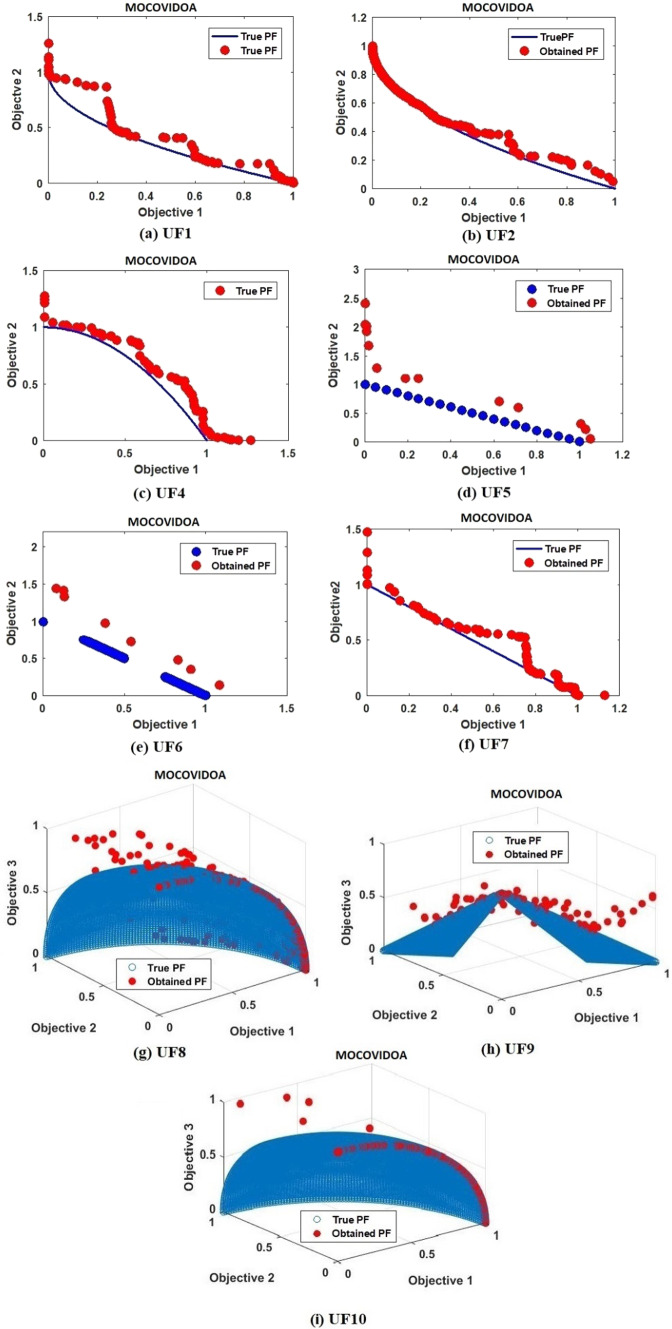
MOCOVIDOA Pareto front for CEC2009 test suit

The CEC2009 test suit is divided into two-objective functions (UF1-UF7) and three-objective functions (UF8-UF10). As shown in Table 3, in the two objective functions, COVIDOA achieves the best results in all evaluation metrics for UF2 and UF4. For the remaining functions, MOCOVIDOA is the best for all metrics in UF1 except the spacing metric, for which MOMVO is the best. In UF3 and UF5 functions, MOEA/D achieves promising results in HV and $\Delta P$ metrics. In UF5 and UF7, MOSMA is superior in terms of HV metric. According to the three-objective functions (UF8-UF10), MOCOVID achieves the best results for UF9. However, MOSMA and MOEA/D achieve the best values of spacing metrics for UF8 and UF10, respectively. The CEC2009 Pareto fronts obtained from running MOCOVIDOA are presented in Fig. 12.

**Table 3:** Results for CEC2009 test suit

Problem	Metric	Algorithm					
		MOEA/D	MOMVO	MOPSO	MOALO	MOSMA	MOCOVIDOA
UF1	IGD	0.21716	0.065442	0.063433	0.08072	0.11177	**0.054127**
	GD	0.009713	0.0076536	0.008562	0.008696	0.033474	**0.004462**
	HV	0.51026	0.61881	0.53221	0.60554	0.55382	**0.054127**
	SP	0.0072095	**0.06216**	0.063738	0.0076305	0.026291	0.010593
	MS	1.228	1.0409	1.35101	1.647	0.9208	**0.8309**
	$\Delta \mathrm{P}$	0.21716	0.073876	0.085471	0.08072	0.33108	**0.055288**
UF2	IGD	0.05166	0.037242	0.057522	0.050013	0.039163	**0.035002**
	GD	0.0046049	0.014877	0.011253	0.06488	0.016726	**0.0042994**
	HV	0.68071	0.67673	0.676511	0.68925	0.68793	**0.67226**
	SP	0.0062903	0.018219	0.044281	0.015431	0.005339	**0.003467**
	MS	1.2933	1.2648	1.22188	1.3131	1.3228	**1.1118**
	$\Delta \mathrm{P}$	0.05166	0.115	0.04548	0.4895	0.092712	**0.036966**
UF3	IGD	0.44493	0.198272	0.211920	0.28734	0.21529	**0.19351**
	GD	0.044506	0.024418	0.023532	0.027641	0.0335351	**0.023052**
	HV	**0.22554**	0.232131	0.232812	0.35742	0.34978	0.22773
	SP	0.22037	0.03122	0.02651	0.028318	0.024106	**0.02026**
	MS	1.7093	1.3425	1.42302	1.3013	2.1465	**1.2244**
	$\Delta \mathrm{P}$	**0.14493**	0.22481	0.24358	0.28734	0.26529	0.19351
UF4	IGD	0.099516	0.064857	0.065523	0.1174	0.11094	**0.058562**
	GD	0.0077704	0.0098809	0.024219	0.011255	0.011653	**0.00322**
	HV	0.35998	0.36808	0.58192	0.37823	0.38354	**0.31486**
	SP	0.012185	0.0176624	0.02815	0.017502	0.01584	**0.011175**
	MS	1.4574	0.93665	0.97811	1.3238	0.96624	**0.66443**
	$\Delta \mathrm{P}$	0.099516	0.079924	0.07739	0.155	0.1477	**0.070676**
UF5	IGD	0.68305	0.33601	0.198202	0.19099	0.43583	**0.19092**
	GD	0.157359	0.15079	0.192616	0.143662	0.25831	**0.14281**
	HV	0.12944	0.25263	0.101671	0.30597	**0.05175**	0.07077
	SP	0.04619	0.018542	0.21277	0.050341	0.1709	**0.0176**
	MS	1.0067	1.0481	1.27413	1.1284	1.5963	**0.8194**
	$\Delta \mathrm{P}$	**0.35869**	0.44741	0.74529	0.44613	1.261	**0.43684**
UF6	IGD	0.30787	0.28424	0.36517	0.73878	1.4038	**0.2864**
	GD	0.35501	0.26795	0.33713	0.23501	0.30232	**0.19854**
	HV	0.14468	0.22923	0.24631	0.042543	0.05244	**0.034978**
	SP	0.4225	0.24906	0.36617	**0.13157**	0.17204	0.39984
	MS	1.57830	1.2027	1.35371	1.2372	0.68435	**0.60604**
	$\Delta \mathrm{P}$	1.63390	0.74452	1.5572	1.7494	4.0543	**1.2268**
UF7	IGD	0.267	0.043946	0.045311	0.082768	0.1797	**0.042834**
	GD	0.01517	0.0044725	0.007782	0.0065911	0.034035	**0.0021517**
	HV	0.3997	0.52295	0.57192	0.46693	**0.3264**	0.40794
	SP	**0.0082483**	0.0057041	0.04312	0.0077955	0.036233	0.035218
	MS	1.0317	1.0589	1.22614	1.471	1.1959	**1.0013**
	$\Delta \mathrm{P}$	0.267	0.043946	0.045591	0.082768	0.3818	**0.040305**
UF8	IGD	0.1329	0.19352	0.13382	0.24352	0.19105	**0.10536**
	GD	0.032258	0.17158	0.18281	0.086732	0.22497	**0.03071**
	HV	0.42664	0.4791	0.4424	0.44534	0.4127	**0.41405**
	SP	0.148358	0.15545	0.12937	0.19863	**0.073247**	0.16392
	MS	1.4588	1.1611	0.78638	0.8273	1.1981	**0.77463**
	$\Delta \mathrm{P}$	0.4391	1.6932	1.4102	0.38628	2.157	**0.36013**
UF9	IGD	0.1196	0.19774	0.14891	0.21679	0.19482	**0.10933**
	GD	0.084191	0.21878	0.22392	0.062314	0.32431	**0.061392**
	HV	0.59328	0.49671	0.57502	0.50075	0.48195	**0.4273**
	SP	0.032234	0.17552	0.27574	0.25872	0.24609	**0.027544**
	MS	1.4761	1.1855	1.09722	1.4985	1.1046	**1.0107**
	$\Delta \mathrm{P}$	0.39711	2.3318	2.2088	0.76422	3.0587	**0.29873**
UF10	IGD	0.237	0.25602	0.23959	0.37393	0.25852	**0.21754**
	GD	0.158567	0.34897	0.36243	0.18822	0.35165	**0.15848**
	HV	0.60894	0.28419	0.32665	0.28011	0.26019	**0.24895**
	SP	**0.057739**	0.16008	1.3943	0.22478	0.14703	0.10799
	MS	1.5162	0.89554	1.0253	1.5676	1.3219	**1.177**
	$\Delta \mathrm{P}$	1.37292	4.1649	3.9481	2.3098	3.9838	**1.3248**

#### Results of constrained test functions

The second group of test functions includes benchmark functions with multiple constraints. A static penalty approach is utilized in the proposed algorithm to handle constraints. The penalty function aims to convert the constrained problem into an unconstrained one by using an artificial penalty for violating the constraint. After calculating the penalty for violating the constraints, this value is added to the fitness function value *f(x)* in the case of minimization problems or subtracted from *f(x)* in the case of maximization problems [48]. The five convergence and coverage metrics mentioned above are utilized to compare all algorithms' performance. Table 4 shows the results of solving the test functions: CONSTR, TNK, BNH, OSY, and SRN. The table shows that the MOCOVIDOA is superior in 21 of 30 cases. In the remaining cases, MOSMA, MOEA/D, and MOMVO slightly outperform MOCOVIDOA, especially in terms of SP and MS metrics.

**Table 4:** Results for TNK, CONSTR, BNH, SRN, and OSY functions

Problem	Metric	Algorithm					
		MOEA/D	MOMVO	MOPSO	MOALO	MOSMA	MOCOVIDOA
CONSTR	IGD	0.22808	0.038448	0.0056	0.052674	0.077501	**0.0012592**
	GD	0.0004866	**0.000331**	0.0098548	0.000768	0.0005900	0.0003682
	HV	0.45331	0.47756	0.43348	0.47592	0.4712	**0.41139**
	SP	**0.013048**	0.042028	0.0346	0.028831	0.095211	0.024507
	MS	1.8703	1.2864	1.6438	1.5281	1.716	**0.85338**
	$\Delta P$	0.22808	0.038448	0.083993	0.052674	0.077501	**0.012592**
TNK	IGD	0.01757	0.009448	0.00887	0.012616	0.021868	**0.0086304**
	GD	0.0009965	0.000588	0.00507	0.0005955	0.0095005	**0.0005805**
	HV	0.38955	0.39384	0.4465	0.38967	**0.37162**	0.38939
	SP	0.010525	**0.007786**	0.7751	0.011838	0.025933	0.096073
	MS	1.1893	0.81924	0.7856	1.0551	0.66801	**0.59754**
	$\Delta P$	0.01757	0.007448	0.0065	0.012616	0.10401	**0.006630**
BNH	IGD	1.3487	1.75	1.534	2.1187	0.33739	**0.21976**
	GD	**0.38738**	1.2898	0.734	2.2065	1.3554	0.54929
	HV	0.69186	0.64032	0.6647	0.66732	0.66379	**0.66359**
	SP	0.91132	0.80305	0.6941	1.7092	0.4667	**0.46516**
	MS	1.339	1.4415	0.8571	2.0421	1.4186	**0.69804**
	$\Delta P$	1.3487	1.6684	1.143	17.7752	0.2181	**0.856**
SRN	IGD	0.0253	0.00454	0.00214	0.001745	0.8454	**0.0014471**
	GD	**0.00356**	0.00231	0.0027	0.00967	0.0334	0.004203
	HV	0.324	0.442	0.734	**0.54318**	0.3326	**0.28855**
	SP	1.344	0.5433	0.6634	0.62257	**0.1846**	**0.245043**
	MS	0.1763	0.2451	0.5832	0.3522	**0.1467**	**0.286511**
	$\Delta P$	0.0066	0.003	0.0024	0.001143	**0.2785**	**0.001429**
OSY	IGD	0.0835	0.0945	0.1043	0.02418	0.0634	**0.010433**
	GD	0.06854	0.0742	0.0845	0.03875	0.0536	**0.034252**
	HV	0.4582	0.3546	0.6634	0.7343	0.442	**0.32968**
	SP	1.534	0.8133	0.5542	0.9379	1.535	**0.80462**
	MS	1.154	0.6845	**0.5793**	0.7827	0.7451	0.66731
	$\Delta P$	0.0765	0.088	0.9956	0.02956	0.05674	**0.02419**

**Fig. 13:**
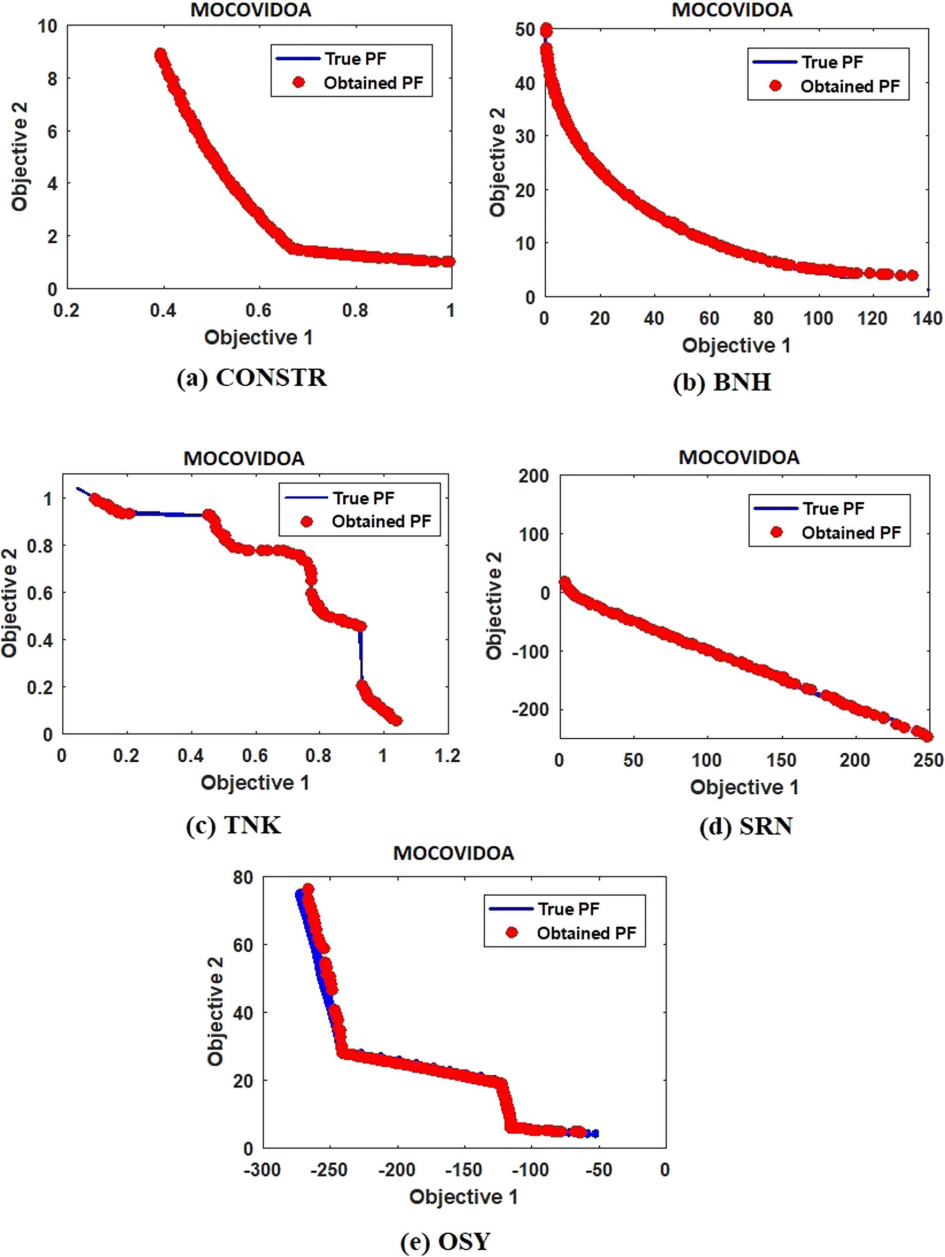
MOCOVIDOA Pareto front for constrained problems: CONSTR, BNH, TNK, SRN, and OSY

The POSs obtained by MOCOVIDOA are presented in Fig. 13; it is obvious from the figure that the constrained test functions have different Pareto fronts, such as the concave front of the CONSTR and the wave-shaped front of the TNK function. The results showed that the proposed MOCOVIDOA could successfully approximate all these fronts. Additionally, the coverage of MOCOVIDOA is clear as the obtained Pareto fronts are well distributed along the true Pareto front.

**Fig. 14:**
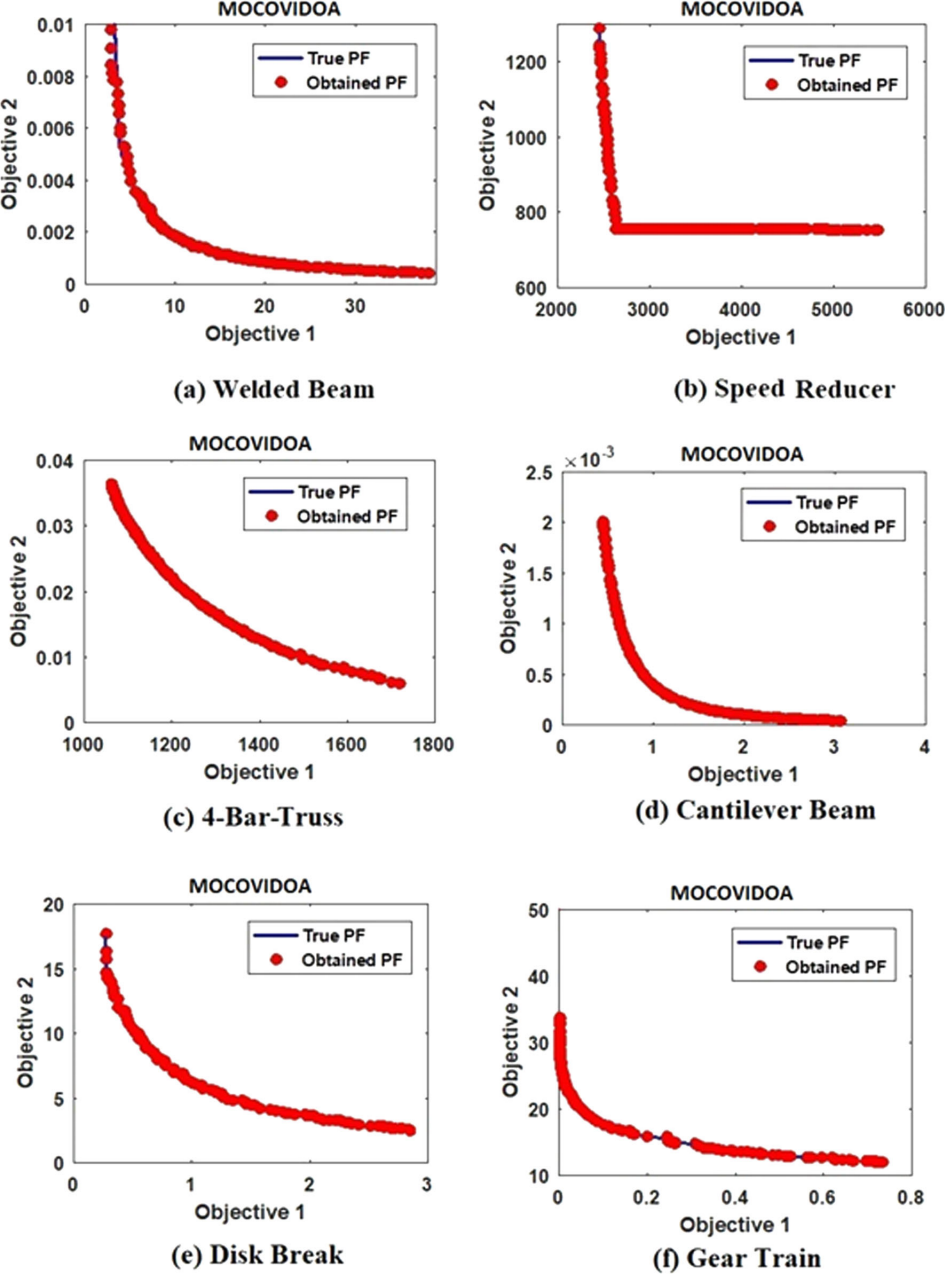
MOCOVIDOA Pareto front for engineering design problems

Finally, the superiority of MOCOVIDOA is supported by utilizing a set of popular real-world engineering design problems. The numerical results for all algorithms are shown in Table 5. Note that, the bold values in the tables indicate the best obtained results. As in the preceding test functions, the proposed MOCOVIDOA shows strong convergence and coverage properties compared to other algorithms. It outperforms other algorithms' peers in 26 of 36 cases. The MOCOVIDOA obtained Pareto fronts for engineering design problems are shown in Fig. 14.

**Table 5:** Results of different engineering design problems

Problem	Algorithm						
	Metric	MOEA/D	MOMO	MOPS	MOALO	MOSMA	MOCOVIDOA
Welded beam	IGD	0.02554	0.00426	0.1653	0.00856	0.0956	**0.00143**
	GD	**0.001254**	0.01442	0.04909	0.00663	0.01467	0.00127
	HV	0.7566	0.7738	0.8301	0.0978	0.0882	**0.08589**
	SP	0.0446	0.05058	0.04257	0.1005	0.16454	**0.04053**
	MS	**0.2631**	0.42711	0.491	0.2253	0.535	**0.18328**
	$\Delta P$	0.0074	0.05672	0.0842	0.00725	**0.00124**	0.01095
Disk break	IGD	0.0957	0.00453	0.7454	0.00243	0.0464	**0.002211**
	GD	0.00126	0.0132	0.06685	0.00332	**0.00102**	0.0032016
	HV	0.08856	1.056	1.2434	1.143	0.08645	**0.085425**
	SP	0.0472	0.2537	.0675	0.0562	0.06543	**0.042712**
	MS	0.0556	0.4482	0.536	0.2201	0.5732	**0.055271**
	$\Delta P$	0.0164	0.0142	0.579	0.00592	0.00837	**0.004206**
Speed reducer	IGD	0.867	0.4482	1.574	0.1635	0.1622	**0.1541**
	GD	1.058	**0.75292**	0.98831	1.0233	0.0754	0.0943
	HV	0.2865	0.3144	0.4534	0.6532	0.2886	**0.2853**
	SP	2.751	1.6354	6.985	0.2242	0.1745	**0.1529**
	MS	1.352	1.033	4.776	1.266	0.6821	**0.0946**
	$\Delta P$	0.9712	0.5643	0.8312	0.6623	0.0846	**0.102**
4-bar-truss	IGD	**0.00467**	0.02563	0.0218	0.758	1.034	0.01145
	GD	0.01857	0.2566	0.3741	0.0425	0.00856	**0.01267**
	HV	**0.2544**	0.445	0.6452	0.5493	0.3231	0.6852
	SP	0.6453	1.3248	2.5303	0.8771	2.153	**0.23522**
	MS	0.6557	0.3428	0.8562	0.7532	0.4679	**0.015528**
	$\Delta P$	0.0039	0.1154	0.0532	0.0845	0.0573	**0.00363**
Gear train	IGD	0.0746	0.00335	0.0784	0.0856	0.0547	**0.003278**
	GD	0.1034	0.05772	0.5377	0.1342	0.1055	**0.05334**
	HV	0.5388	0.0574	0.7452	0.5863	**0.0142**	0.04897
	SP	0.2424	0.0967	0.4351	0.2265	0.08224	**0.06652**
	MS	1.0543	0.9671	1.365	1.327	0.86259	**0.42871**
	$\Delta P$	0.0968	0.0217	0.214	1.214	0.0253	**0.01528**
Cantilever beam	IGD	0.00175	0.00064	0.1327	0.000186	0.00673	**0.000153**
	GD	**0.00028**	0.000825	0.00553	0.005367	0.00167	0.0007254
	HV	0.6351	0.3522	0.7113	0.0538	0.0253	**0.0332**
	SP	0.0256	0.00865	0.04427	0.00832	0.01534	**0.00822**
	MS	0.9576	1.324	1.167	0.76731	**0.4163**	0.6736
	$\Delta P$	0.0017	0.000745	0.0564	0.000225	0.00165	**0.000216**

The superior coverage of MOCOVIDOA is due to the COVID selection and archive mechanism, which includes exploring the uncovered areas of the search space by selecting solutions from less-populated neighborhoods from the archive and discarding solutions in the most crowded regions.

The proposed MOCOVIDOA uses the same updating mechanism as COVIDOA, based on the frameshifting technique for replication and mutation. Using the Frameshifting technique and mutation process with an appropriate mutation rate helps the proposed algorithm find the most promising region of the search space and converge to the set of POSs.

#### Sensitivity analysis

This section introduces a study about the sensitivity of MOCOVIDOA performance to different parameters as follows:

A. A maximum number of iterations: We ran the proposed MOCOVIDOA using 100, 200, and 500 iterations to assess the impact of the number of iterations on MOCOVIDOA performance. The GD results of all cases displayed in Table 6 indicate that a maximum number of iterations of 200 yields the best results in most cases.
B. Population size: A population size of 100, 200, and 500 is used to test the sensitivity of MOCOVIDOA to the number of solutions in the population. According to Table 7, the performance of MOCOVIDOA is better with the rise in population size. The results of 200 and 500 population sizes are close to each other; however, a population size of 200 would be preferable to reduce the algorithm's running time.
C. Mutation rate (MR): The mutation rate greatly impacts the performance of MOCOVIDOA. We used three values for MR as follows: 0.5, 0.05, and 0.005, and we display the results in Table 8. It is shown from the table that an MR of 0.05 achieves the best GD values, which indicates that the value of MR should not be very high or very low to make a good balance between exploration and exploitation properties.

**Table 6:** Sensitivity analysis of a maximum number of iterations

No. of Iterations	Problem					
	ZDT1	ZDT2	ZDT3	ZDT4	ZDT6	ZDT1LP
**100**	9.8258e-05	4.9748e-05	0.00041621	0.0018935	0.00063951	0.00029393
**200**	**8.9626e-05**	**3.0389e-05**	**0.00036273**	0.00026446	**0.00038831**	**0.00027269**
**500**	0.00014155	3.583e-05	0.0004008	**0.00017481**	0.00054551	0.00029002

**Table 7:** Sensitivity analysis of population size

Population size	Problem					
	ZDT1	ZDT2	ZDT3	ZDT4	ZDT6	ZDT1LP
100	0.00017571	4.2495e–05	0.0004438	0.0018112	0.0010844	0.00042015
200	8.2268e-05	**3.2759e–05**	**0.00022583**	0.00049255	**2.6561e–05**	0.00029488
500	**6.3781e-05**	5.0018e–05	0.00028684	**0.00031791**	0.00021716	**0.00017586**

**Table 8:** Sensitivity analysis of mutation rate (MR)

Mutation Rate (MR)	Problem					
	ZDT1	ZDT2	ZDT3	ZDT4	ZDT6	ZDT1LP
**0.5**	9.932e–05	7.1191e–05	0.0003557	0.0004349	0.00083803	0.00028813
**0.05**	**7.7289e–05**	**3.3809e–05**	**0.00030485**	**0.00027899**	**0.00051371**	0.00028646
**0.005**	0.00011036	4.0278e–05	0.00035557	0.00038636	0.00076678	**0.00027678**

## Conclusion

This paper proposed the multi-objective version of the recently proposed COVIDOA called MOCOVIDOA, which aims to solve optimization problems with up to three objectives. It maintains the search mechanism of COVIDOA and an archiving concept and selection mechanism. The solutions in MOCOVIDOA are evaluated by using the Pareto optimal dominance operator. The proposed algorithm tests 27 test functions, including 16 unconstrained, five constrained, and six real-world engineering design problems. The performance is evaluated using different metrics, including IGD, GD, SP, HV, MS, and $\Delta P$, presenting the best Pareto front obtained by the proposed algorithm. The superiority of the proposed algorithm is demonstrated by comparing it to five well-regarded multi-objective techniques: MOSMA, MOALO, MOPSO MOMVO, and MOEA/D. The results showed that the proposed MOCOVIDOA outperforms the other techniques in most test cases and its high convergence and coverage capabilities. Using test functions with different Pareto fronts demonstrates the ability of MOCOVIDOA to find different shapes of Pareto fronts. Finally, the results of solving different engineering problems demonstrate the capability of MOCOVIDOA to solve real-world multi-objective problems with multiple conflicting constraints.

## Data Availability

The datasets generated during and/or analyzed during the current study are available from the corresponding author on reasonable request.

## Appendix A: Abbreviation

**Table:**

A novel multi-objective coronavirus disease optimization algorithm	MOCOVIDOA
Inverted generational distance	IGD
Generational distance	GD
Maximum spread	MS
Spacing	SP
Hyper volume	HV
Genetic algorithm	GA
Ant colony optimization	ACO
Differential evolution	DE
Cuckoo search algorithm	CSA
Particle swarm optimization	PSO
Non-dominated sorting genetic algorithm	NSGA
Multi-objective particle swarm optimization	MOPSO
Multi-objective evolutionary algorithm based on decomposition	MOEA/D
Pareto archived evolution strategy	PAES
Multi-objective slime mold algorithm	MOSMA
Multi-objective jellyfish search	MOJS
Multi-objective marine predator algorithm	MOMPA
Multi-objective volleyball premier league algorithm	MOVPA
Multi-objective seagull optimization algorithm	MOSOA
Coronavirus optimization algorithm	COVIDOA
Pareto optimal solutions	POSs
No free lunch	NFL

## Appendix B

Unconstrained multi-objective test problems. See Tables 9 and 10.

**Table 9:** ZDT test suite

ZDT1	
Minimize:	$f_{1} \left( t \right) = t_{1}$
Minimize:	$f_{2} \left( t \right) = g\left( t \right)~ \times h\left( {f_{1} \left( t \right),~g\left( t \right)} \right)$
Where:	$G\left( t \right) = 1 + \frac{9}{{N - 1}}\mathop \sum \limits_{{i = 2}}^{N} t_{i}$
	$${h }({f}1 ({t}),{ g }({t})) = 1 - \sqrt{\frac{{f}1 ({t})}{{g }({t})}}$$
	$$0 \le { ti }\le 1$$
	$$1 \le { i }\le 30$$
ZDT2	
Minimize:	${{f}}_{1}\left({t}\right)={{t}}_{1}$
Minimize:	${{f}}_{2}\left({t}\right)={g}\left({t}\right) \times {h}({{f}}_{1}\left({t}\right),{ g}({t}))$
Where:	${G}\left({x}\right)=1+\frac{9}{{N}-1}\sum_{{i}=2}^{{N}}{{t}}_{{i}}$
	$${h }({f}1 ({t}),{ g }({t})) = 1 {-(\frac{{f}1 ({t})}{{g }({t})})}^{2}$$
	$$0 \le { ti }\le 1$$
	$$1 \le { i }\le 30$$
ZDT3	
Minimize:	${{f}}_{1}\left({t}\right)={{t}}_{1}$
Minimize:	${{f}}_{2}\left({t}\right)={g}\left({x}\right) \times {h}({{f}}_{1}\left({t}\right),{ g}({t}))$
Where:	${G}\left({t}\right)=1+\frac{9}{29}\sum_{{i}=2}^{{N}}{{t}}_{{i}}$
	$${h }\left({f}1 \left({t}\right),{ g }\left({t}\right)\right)= 1-\sqrt{\frac{{f}1 \left({t}\right)}{{g }\left({t}\right)}}-\left(\frac{{f}1 \left({t}\right)}{{g }\left({t}\right)}\right){sin}(10\uppi {{f}}_{1}({t}))$$
	$$0 \le { ti }\le 1$$
	$$1 \le { i }\le 30$$
ZDT4	
Minimize:	${{f}}_{1}\left({t}\right)={{t}}_{1}$
Minimize:	${{f}}_{2}\left({t}\right)={g}\left({t}\right) \times {h}({{f}}_{1}\left({t}\right),{ g}({t}))$
Where:	${G}\left({t}\right)=91+\sum_{{i}=2}^{10}({{t}}_{{i}}^{2}-10*{cos}(4\uppi {{t}}_{{i}}))$
	$${h }\left({f}1 \left({t}\right),{ g }\left({t}\right)\right)= 1-\sqrt{\frac{{f}1 \left({t}\right)}{{g }\left({t}\right)}}$$
	$$0 \le { ti }\le 1$$
	$$1 \le { i }\le 30$$
ZDT6	
Minimize:	${{f}}_{1}\left({t}\right)=1-{{e}}^{\left(-4{{t}}_{1}\right)}{{sin}}^{6}(6\uppi {{t}}_{1})$
Minimize:	${{f}}_{2}\left({t}\right)={g}\left({t}\right)\left[1-{(\frac{{f}1 \left({t}\right)}{{g }\left({t}\right)})}^{2}\right]$
Where:	$G\left( t \right) = 1 + 9\left[ {\frac{{\mathop \sum \nolimits_{{i = 2}}^{n} t_{i} }}{{n - 1}}} \right]^{{\frac{1}{4}}}$
	$$0 \le { ti }\le 1$$
	$$1 \le { i }\le 30$$
ZDT1 with linear PF:	
Minimize:	${{f}}_{1}\left({t}\right)={{t}}_{1}$
Minimize:	${{f}}_{2}\left({t}\right)={g}\left({t}\right) \times {h}({{f}}_{1}\left({t}\right),{ g}({t}))$
Where:	${G}\left({t}\right)=1+\frac{9}{{N}-1}\sum_{{i}=2}^{{N}}{{t}}_{{i}}$
	$${h }({f}1 ({t}),{ g }({t})) = 1 - \frac{{f}1 ({t})}{{g }({t})}$$
	$$0 \le { ti }\le 1$$
	$$1 \le { i }\le 30$$

**Table 10:** CEC2009 Test problems

UF1	
Minimize:	${f}_{1}= {x}_{1}+ \frac{2}{\left|{J}_{1}\right|}{\sum }_{j\epsilon {J}_{1}}{\left[{x}_{j}-{sin}(6\pi {x}_{1}+\frac{j\pi }{n})\right]}^{2}$
Minimize:	${f}_{2}=1-\sqrt{x}+\frac{2}{\left|{J}_{1}\right|} {\sum }_{j\epsilon {J}_{2}}{\left[{x}_{j}-{sin}(6\pi {x}_{1}+\frac{j\pi }{n})\right]}^{2}$
Where:	${{J}}1{ } = { }\left\{ {{\text{j }}\left| {{\text{j is odd }}\&\, { }2{ } \le {\text{ j }} \le {\text{ n}}} \right.{ }} \right\}$
	$${{J}}2{ } = { }\left\{ {{{j }}\left| {{\text{j is even }}\&\, { }2{ } \le {{ j }} \le {{ n}}} \right.{ }} \right\}$$
UF2	
Minimize:	${f}_{1}= {x}_{1}+ \frac{2}{\left|{J}_{1}\right|}{\sum }_{j\epsilon {J}_{1}}{y}_{j}^{2}$
Minimize:	${f}_{2}=1-\sqrt{x}+\frac{2}{\left|{J}_{1}\right|} {\sum }_{j\epsilon {J}_{2}}{y}_{j}^{2}$
Where:	${{J}}1{ } = { }\left\{ {{{j }}\left| {{\text{j is odd }}\& { }2{ } \le {{ j }} \le {{ n}}} \right.{ }} \right\}$
	$${{J}}2{ } = { }\left\{ {{{j }}\left| {{\text{j is even }}\& { }2{ } \le {{ j }} \le {{ n}}} \right.{ }} \right\}$$
	$${y}_{j}=\left\{\begin{array}{c}{x}_{j}-\left[0.3 {x}_{1}^{2}{cos}\left(24\pi {x}_{1}+\frac{4j\pi }{n}\right) + 0.6 {x}_{1}\right]{cos}\left(6\pi {x}_{1}+\frac{j\pi }{n}\right), if j\epsilon {J}_{1} \\ {x}_{j}-\left[0.3 {x}_{1}^{2}{cos}\left(24\pi {x}_{1}+\frac{4j\pi }{n}\right) + 0.6 {x}_{1}\right]{sin}\left(6\pi {x}_{1}+\frac{j\pi }{n}\right), if j\epsilon {J}_{2}\end{array}\right.$$
UF3	
Minimize:	$f_{1} = x_{1} + \frac{2}{{\left| {J_{1} } \right|}}\left( {4 \mathop \sum \limits_{{j\epsilon J_{1} }} y_{j}^{2} - 2 \mathop \prod \limits_{{j\epsilon J_{1} }} \cos \left( {\frac{{20 y_{j} \pi }}{\sqrt j }} \right) + 2} \right)$
Minimize:	${f}_{2}= \sqrt{{x}_{1}}+ \frac{2}{\left|{J}_{1}\right|}(4 {\sum }_{j\epsilon {J}_{1}}{y}_{j}^{2}-2 {\prod }_{j\epsilon {J}_{2}}{cos}\left(\frac{20 {y}_{j}\pi }{\sqrt{j}}\right)+2)$
Where:	${{J}}1{ } = { }\left\{ {{{j }}\left| {{\text{j is odd }}\&\, { }2{ } \le {{ j }} \le {{ n}}} \right.{ }} \right\}$
	$${{J}}2{ } = { }\left\{ {{{j }}\left| {{\text{j is even }}\&\, { }2{ } \le {{ j }} \le {{ n}}} \right.{ }} \right\}$$
	$${y}_{j}={x}_{j}- {x}_{1}^{0.5\left(1.0+\frac{3\left(j-2\right)}{n-2}\right)}, j=2, 3,\dots ,n$$
UF4	
Minimize:	${f}_{1}= {x}_{1}+ \frac{2}{\left|{J}_{1}\right|}{\sum }_{j\epsilon {J}_{1}}h({y}_{j})$
Minimize:	${f}_{2}=1-{x}_{2}+\frac{2}{\left|{J}_{1}\right|} {\sum }_{j\epsilon {J}_{2}}h({y}_{j})$
Where:	${{J}}1{ } = { }\left\{ {{{j }}\left| {{\text{j is odd }}\& { }2{ } \le {{ j }} \le {{ n}}} \right.} \right\}$
	$${{J}}2{ } = { }\left\{ {{{j }}\left| {{\text{j is even }}\& { }2{ } \le {{ j }} \le {{ n}}} \right.} \right\}$$
	$${y}_{j}={x}_{j}-{sin}\left(6\pi {x}_{1}+\frac{j\pi }{n}\right), j=2, 3,\dots ,n, h\left(t\right)=\frac{\left|t\right|}{1+{e}^{2\left|t\right|}}$$
UF5	
Minimize:	${f}_{1}= {x}_{1}+\left( \frac{1}{2N}+\epsilon \right)\left|{sin}\left(2N\mu {x}_{1}\right)\right|+\frac{2}{\left|{J}_{1}\right|}{\sum }_{j\epsilon {J}_{1}}h({y}_{j})$
Minimize:	${f}_{2}={1-x}_{1}+\left( \frac{1}{2N}+\epsilon \right)\left|{sin}\left(2N\mu {x}_{1}\right)\right|+\frac{2}{\left|{J}_{2}\right|}{\sum }_{j\epsilon {J}_{2}}h({y}_{j})$
Where:	${{J}}1{ } = { }\left\{ {{{j }}\left| {{\text{ j is odd }}\& { }2{ } \le {{ j }} \le {{ n}}} \right.} \right\}$
	$${{J}}2{ } = { }\left\{ {{{j }}\left| {{{j is even }}\& { }2{ } \le {{ j }} \le {{ n}}} \right.{ }} \right\}$$
	$$\epsilon >0, { y}_{j}={x}_{j}-{sin}\left(6\pi {x}_{1}+\frac{j\pi }{n}\right), j=2, 3,\dots ,n, h\left(t\right)=2{t}^{2}-{cos}(4\pi t+1)$$
UF6	
Minimize:	${f}_{1}= {x}_{1}+{max}\{{0,2}\left( \frac{1}{2N}+\epsilon \right){sin}\left(2N\mu {x}_{1}\right)\}+\frac{2}{\left|{J}_{1}\right|}(4{\sum }_{j\epsilon {J}_{1}}{y}_{j}^{2}$ $-2 {\prod }_{j\epsilon {J}_{1}}{cos}\left(\frac{20 {y}_{j}\pi }{\sqrt{j}}\right)+1)$
Minimize:	${f}_{2}= 1-{x}_{1}+{max}\{{0,2}\left( \frac{1}{2N}+\epsilon \right){sin}\left(2N\mu {x}_{1}\right)\}+\frac{2}{\left|{J}_{2}\right|}(4{\sum }_{j\epsilon {J}_{2}}{y}_{j}^{2}$ $-2 {\prod }_{j\epsilon {J}_{2}}{cos}\left(\frac{20 {y}_{j}\pi }{\sqrt{j}}\right)+1)$
Where:	${{J}}1{ } = { }\left\{ {{{j}}\left| {{\text{j isj odd }}\& { }2{ } \le {{ j }} \le {{ n}}} \right.} \right\}$
	$${{J}}2{ } = { }\left\{ {{{j }}\left| {{\text{j isj even }}\& { }2{ } \le {{ j }} \le {{ n}}} \right.} \right\}$$
	$$\epsilon >0, { y}_{j}={x}_{j}-{sin}\left(6\pi {x}_{1}+\frac{j\pi }{n}\right), j=2, 3,\dots ,n$$
UF7	
Minimize:	${f}_{1}= \sqrt[5]{{x}_{1}}+\frac{2}{\left|{J}_{1}\right|}{\sum }_{j\epsilon {J}_{1}}{y}_{j}^{2}$
Minimize:	${f}_{2}=1- \sqrt[5]{{x}_{1}}+\frac{2}{\left|{J}_{2}\right|}{\sum }_{j\epsilon {J}_{2}}{y}_{j}^{2}$
Where:	${{J}}1{ } = { }\left\{ {{{j }}\left| {{\text{j is odd }}\& { }2{ } \le {{ j }} \le {{ n}}} \right.{ }} \right\}$
	$${{J}}2{ } = { }\left\{ {{{j }}\left| {{\text{j isj even }}\& { }2{ } \le {{ j }} \le {{ n}}} \right.} \right\}$$
	$$\epsilon >0, { y}_{j}={x}_{j}-{sin}\left(6\pi {x}_{1}+\frac{j\pi }{n}\right), j=2, 3,\dots ,n$$
UF8	
Minimize:	${f}_{1}={cos}\left(0.5{x}_{1}\pi \right){cos}\left(0.5{x}_{2}\pi \right)+\frac{2}{\left|{J}_{1}\right|}{\sum }_{j\epsilon {J}_{1}}({x}_{j}-2{x}_{2} {sin}{(2\pi {x}_{1}+\frac{j\pi }{n})}^{2})$
Minimize:	${f}_{2}={cos}\left(0.5{x}_{1}\pi \right){cos}\left(0.5{x}_{2}\pi \right)+\frac{2}{\left|{J}_{2}\right|}{\sum }_{j\epsilon {J}_{2}}({x}_{j}-2{x}_{2} {sin}{(2\pi {x}_{1}+\frac{j\pi }{n})}^{2})$
Minimize:	${f}_{3}={sin}\left(0.5{x}_{1}\pi \right)+\frac{2}{\left|{J}_{3}\right|}{\sum }_{j\epsilon {J}_{3}}({x}_{j}-2{x}_{2} {sin}{(2\pi {x}_{1}+\frac{j\pi }{n})}^{2})$
Where:	$J_{1} = \left\{ {j \left| { 3 \le j \le n, } \right. {{and}} j - 1 {\text{is\, a\, multiplication \,of}} 3} \right\}$
	$$J_{2} = \left\{ {j \left| { 3 \le j \le n, } \right.{{and}} j - 2 {\text{is\, a\, multiplication\, of}} 3} \right\}$$
	$${J}_{3}=\left\{j \left| 3\le j\le n, \right. and\, j\, is\, a\, multiplication\, of\, 3\right\}, \epsilon =0.1$$
UF9	
Minimize:	${f}_{1}=0.5 \left[{max}\left\{0,\left(1+\epsilon \right)\left(1-4{\left(2{x}_{1}-1\right)}^{2}\right)\right\}+2{x}_{1}\right]{x}_{2}+\frac{2}{\left|{J}_{1}\right|}{\sum }_{j\epsilon {J}_{1}}({x}_{j}-2{x}_{2} {sin}{(2\pi {x}_{1}+\frac{j\pi }{n})}^{2})$
Minimize:	${f}_{2}=0.5 \left[{max}\left\{0,\left(1+\epsilon \right)\left(1-4{\left(2{x}_{1}-1\right)}^{2}\right)\right\}+2{x}_{1}\right]{x}_{2}+\frac{2}{\left|{J}_{2}\right|}{\sum }_{j\epsilon {J}_{2}}({x}_{j}-2{x}_{2} {sin}{(2\pi {x}_{1}+\frac{j\pi }{n})}^{2})$
Minimize:	${f}_{3}=1- {x}_{2}+ \frac{2}{{J}_{3}}{\sum }_{j\epsilon {J}_{2}}({x}_{j}-2{x}_{2} {sin}{(2\pi {x}_{1}+\frac{j\pi }{n})}^{2})$
Where:	${J}_{1}=\{j \left| 3\le j\le n, \right. and j-1 is a multiplication of 3\}$
	$${J}_{2}=\{j \left| 3\le j\le n, \right. and j-2 is a multiplication of 3\}$$
	$${J}_{3}=\left\{j \left| 3\le j\le n, \right. and j is a multiplication of 3\right\}, \epsilon =0.1$$
UF10	
Minimize:	${f}_{1}={cos}\left(0.5{x}_{1}\pi \right){cos}\left(0.5{x}_{2}\pi \right)+\frac{2}{\left|{J}_{1}\right|}{\sum }_{j\epsilon {J}_{1}}({4y}_{i}^{2}-{cos}\left(8\pi {y}_{i}\right)+1)$
Minimize:	${f}_{2}={cos}\left(0.5{x}_{1}\pi \right){cos}\left(0.5{x}_{2}\pi \right)+\frac{2}{\left|{J}_{2}\right|}{\sum }_{j\epsilon {J}_{2}}({4y}_{i}^{2}-{cos}\left(8\pi {y}_{i}\right)+1)$
Minimize:	${f}_{3}={sin}\left(0.5{x}_{1}\pi \right)+\frac{2}{\left|{J}_{3}\right|}{\sum }_{j\epsilon {J}_{3}}({x}_{j}-2{x}_{2} {sin}{(2\pi {x}_{1}+\frac{j\pi }{n})}^{2})$
Where:	$J_{1} = \left\{ {j \left| { 3 \le j \le n, }\, \right.\ and\ j - 1{\text{is\, a\, multiplication\, of}}\, 3} \right\}$
	$$J_{2} = \left\{ {j \left| { 3 \le j \le n, }\, \right.and\,\, j - 2{\text{ is a multiplication of}}\,\, 3} \right\}$$
	$$J_{3} = \left\{ {j \left| { 3 \le j \le n, } \right.{\text{ and j is a multiplication of}} 3} \right\}, \epsilon = 0.1$$

## Appendix C

Constrained multi-objective test problems utilized in this work

See Table 11

**Table 11:** CONSTR, BNH, TNK, SRN, and OSY problems

CONSTR	
Minimize:	${{f}}_{1}\left({t}\right)={{t}}_{1}$
Minimize:	${{f}}_{2}\left({t}\right)=(1+{{t}}_{2})/{{t}}_{1}$
Where:	${{G}}_{1}\left({t}\right)=6-({{t}}_{2}+9 {{t}}_{1})$
	$${{G}}_{2}\left({t}\right)=1+{{t}}_{2}-9 {{t}}_{1}$$
	$$0 \le {t}_{1} \le 1$$
	$$0 \le {t}_{2}\le 5$$
BNH	
Minimize:	${{f}}_{1}\left({t}\right)={4{t}}_{1}^{2}+ {4{t}}_{2}^{2}$
Minimize:	${{f}}_{2}\left({t}\right)={({t}_{1}-5)}^{2}+{({t}_{2}-5)}^{2}$
Where:	${{G}}_{1}\left({t}\right)={({t}_{1}-5)}^{2}+{{t}}_{2}^{2}-25$
	$${{G}}_{2}\left({t}\right)=17.7-{({t}_{1}-8)}^{2}-{({t}_{2}+3)}^{2}$$
	$$0 \le {{t}}_{1} \le 5$$
	$$0 \le {{t}}_{2}\le 3$$
TNK	
Minimize:	${{f}}_{1}\left({t}\right)={{t}}_{1}$
Minimize:	${{f}}_{2}\left({t}\right)={{t}}_{2}$
Where:	${{G}}_{1}\left({t}\right)={-{t}}_{1}^{2}{-{t}}_{2 }^{2}+1+0.1{cos}(16{arctan}(\frac{{t}_{1}}{{t}_{2}}))$
	$${{G}}_{1}\left({t}\right)=0.5-{\left({t}_{1}-0.5\right)}^{2} -{({t}_{2}-0.5)}^{2}$$
	$$0.1\le {{t}}_{1} \le\uppi $$
	$$0 \le {{t}}_{2}\le\uppi $$
SRN	
Minimize:	${{f}}_{1}\left({t}\right)=2+{({{t}}_{1}-2)}^{2}+{({{t}}_{2}-1)}^{2}$
Minimize:	${{f}}_{2}\left({t}\right)=9{{t}}_{1}-{({{t}}_{2}-1)}^{2}$
Where:	${{G}}_{1}\left({t}\right)={t}_{1}^{2}+ {t}_{2}^{2}-255$
	$${{G}}_{2}\left({t}\right)={{t}}_{1}-3{{t}}_{2}+10$$
	$$-20\le {{t}}_{1} \le 20$$
	$$-20 \le {{t}}_{2}\le 20$$
OSY	
Minimize:	${{f}}_{1}\left({t}\right)={t}_{1}^{2}+ {t}_{2}^{2}+{t}_{3}^{2}+{t}_{4}^{2}+{t}_{5}^{2}+{t}_{6}^{2}$
Minimize:	${{f}}_{2}\left({t}\right)=-[25 {\left({t}_{1}-2\right)}^{2}+{\left({t}_{2}-1\right)}^{2}+ {\left(3-1\right)}^{2}+{\left({t}_{4}-4\right)}^{2}+{\left({t}_{5}-1\right)}^{2}$
Where:	${{G}}_{1}\left({t}\right)=2- {t}_{1}- {t}_{2}$
	$${{G}}_{2}\left({t}\right)=-6+ {t}_{1}+ {t}_{2}$$
	$${{G}}_{3}\left({t}\right)=-2- {t}_{1}+ {t}_{2}$$
	$${{G}}_{4}\left({t}\right)=-2+ {t}_{1}- 3{t}_{2}$$
	$${{G}}_{5}\left({t}\right)=-4+ {t}_{4}+{({t}_{3}-3)}^{2}$$
	$${{G}}_{5}\left({t}\right)=4- {t}_{6}-{({t}_{5}-3)}^{2}$$
	$$0\le {{x}}_{1,}{x}_{2} \le 10$$
	$$1\le {{x}}_{3,}{{x}}_{5}\le 5$$
	$$0\le {x}_{4}\le 6$$
	$$0\le {x}_{6}\le 1$$
Welded beam design problem	
Minimize:	${{f}}_{1}\left({t}\right)=1.10471*{{t}\left(1\right)}^{2}*t\left(2\right)+0.04811*t\left(3\right)*t\left(4\right)*(14.0+t(2))$
Minimize:	${{f}}_{2}\left({t}\right)=65856000/(30 * {10}^{6} * t(4) * {t(3)}^{3})$
Where:	${G}_{1}(t)= \tau -13600$
	$${G}_{2}(t)= \sigma -30000$$
	$${G}_{3}\left(t\right)= t\left(1\right)-t(4)$$
	$${G}_{4}\left(t\right)= 6000-P$$
	$$0.125 \le t1 \le 5$$
	$$0.1 \le t2 \le 10$$
	$$0.1 \le t3 \le 10$$
	$$0.125 \le t4 \le 5$$
	$$q=6000+\left(14+\frac{t\left(2\right)}{2}\right), D=sqrt(\frac{{t(2)}^{2}}{4}+\frac{(t\left(1\right)+{t(3)}^{2})}{4})$$
	$$J=2*(t\left(1\right)*t\left(2\right)*sqrt\left(2\right)*(\frac{{t(2)}^{2}}{12}+\frac{(t\left(1\right)+{t(3)}^{2})}{4}))$$
	$$\alpha =\frac{6000}{sqrt\left(2\right)*t\left(1\right)*t\left(2\right)}$$
	$$\beta =Q*\frac{D}{J}$$
	$$\tau =sqrt\left({\alpha }^{2}+2* \alpha *\beta *\frac{t\left(2\right)}{2*D}+{\beta }^{2}\right)$$
	$$\sigma =\frac{504000}{t\left(4\right)*{t(3)}^{2}}$$
	$$tmpf=4.013*\frac{30*{10}^{6}}{196}$$
	$$P=tmpf*sqrt\left({t\left(3\right)}^{2}*\frac{{t\left(4\right)}^{6}}{36}\right)*(1-t\left(3\right)*\frac{sqrt(\frac{30}{48})}{28})$$
Cantilever beam design problem	
Minimize:	${f}_{1}(t) = 0.25 * \rho * \pi * t(2) * {t(1) }^{2}$
Minimize:	${f}_{2}(t) = (64 * P * {t(2)}^{3} )/(3 * E * \pi * {t(1)}^{4})$
Where:	${G}_{1}(t) = -Sy + (32 * P * t(2))/(\pi * {t(1)}^{3} )$
	$${G}_{2}(t) = -\delta max + (64 * P * {t(2)}^{3})/(3 * E * \pi * {t(1) }^{4})$$
	$$0.01 \le t1 \le 0.05$$
	$$0.20 \le t2 \le 1$$
	$$P =1, E =207000000, Sy =300000, \delta max =0.005; \rho =7800$$
Speed reducer design problem	
Minimize:	$${f}_{1}\left(t\right)= 0.7854*t\left(1\right)*{t\left(2\right)}^{2}*\left(3.3333 * {t\left(3\right)}^{2} +14.9334 * t\left(3\right)\right)\dots $$ $$43.0934) - 1.508 *{ t}(1) * ({{t}(6)}^{2} +{{ t}(7)}^{2}$$
	$$+ 7.4777 * \left(t\left(6\right)3 + t\left(7\right)3\right)\dots $$
	$$+ 0.7854 * (t(4) * t(6) 2 + t(5) * t(7) 2)$$
Minimize:	${f}_{2}(t) = ((sqrt {(((745 * t(4))/t(2) * t(3)))}^{2} +19.9e6))/(0.1 *{ t(6)}^{3} ))$
Where:	${G}_{1}\left(t\right)=\frac{27}{t\left(1\right)* {t\left(2\right)}^{2} * t\left(3\right)}- 1$
	$${G}_{2}\left(t\right)=\frac{397.5}{t\left(1\right)* {t\left(2\right)}^{2} * {t\left(3\right)}^{2}}- 1$$
	$${G}_{3}(t) = (1.93 *\frac{{t\left(4\right)}^{3} }{t\left(2\right)* t\left(3\right)* {t\left(6\right)}^{4} }- 1$$
	$${G}_{4}(t) = (1.93 * ({t(5)}^{3} )/(t(2) * t(3) * {t(7)}^{4} ) - 1$$
	$${G}_{5}(t) = (\left(sqrt\frac{\left({(745 * t(4))/(t(2) * t(3)))}^{2} + 16.9e6\right)}{110 * {t\left(6\right)}^{3} }\right)- 1$$
	$${G}_{6}(t) = (\left(sqrt\frac{\left({(745 * t(5))/(t(2) * t(3)))}^{2} + 157.5e6\right)}{85 * {t\left(7\right)}^{3} }\right)- 1$$
	$${G}_{7}\left(t\right)= \left(\frac{t\left(2\right)* t\left(3\right)}{40}\right)- 1$$
	$${G}_{8}\left(t\right)= \left(5 *\frac{t\left(2\right)}{t\left(1\right)}\right)- 1$$
	$${G}_{9}\left(t\right)= \left(\frac{t\left(1\right)}{12}* t\left(2\right)\right)- 1$$
	$${G}_{10}\left(t\right)= \left(\frac{1.5 * t\left(6\right)+ 1.9}{t\left(4\right)}\right)- 1$$
	$${G}_{11}(t) = ((1.1 * t(7) + 1.9)/t(5)) - 1$$
	$$2.6 \le t1 \le 3.6$$
	$$0.7 \le t2 \le 0.8$$
	$$17 \le t3 \le 28$$
	$$7.3 \le t4 \le 8.3$$
	$$7.3 \le t5 \le 8.3$$
	$$2.9 \le t6 \le 3.9$$
	$$5 \le t7 \le 5.5$$
Four-bar truss design problem	
Minimize:	${f}_{1}(t) = 200 * (2 * t(1) + sqrt(2 * t(2)) + sqrt (t(3)) + t(4))$
Minimize:	${f}_{2}(t) = 0.01 * ( \left(\frac{2}{t\left(1\right)}\right)+\left(\frac{2*sqrt\left(2\right)}{t\left(2\right)}\right)-\left(\frac{2*sqrt\left(2\right)}{t\left(3\right)}\right)\dots$
	$$- ((2 * sqrt(2))/t(3)) + (2/t(1)))$$
Where:	$1\le t1 \le 3$
	$$1.4142 \le t2 \le 3$$
	$$1.4142 \le t3 \le 3$$
	$$1 \le t4 \le 3$$
Disk brake design problem	
Minimize:	${f}_{1}(t) = 4.9 * (10(-5) ) * (t(2) 2-t(1) 2) * (t(4)-1)$
Minimize:	${f}_{2}(t) = (9.82 * (10(6) )) * (t(2) 2 - t(1) 2))/((t(2) 3 - t(1) 3) * t(4) * t(3))$
Where:	${G}_{1}(t) = 20 + t(1) - t(2)$
	$${G}_{2}(t) = 2.5 * ({t}(4) + 1) - 30$$
	$${G}_{3}(t) = ({t}(3))/(3.14 * {({{t}(2) }^{2} - {{t}(1)}^{2}) }^{2}) - 0.4$$
	$${G}_{4}(t) = (2.22 * {10}^{-3} *{ t}(3) * ({{t}(2)}^{3} - {{t}(1)}^{3}))/({({{t}(2)}^{2} - {{t}(1)}^{2} )}^{2}) - 1$$
	$${G}_{5}(t) = = 900 - (2.66 * {10}^{-2} *{ t}(3) *{ t}(4) * ({{t}(2)}^{3} - {{t}(1)}^{3} ))/(({{t}(2)}^{2} - {{t}(1) }^{2}))$$
	$$55\le t1 \le 80$$
	$$75\le t2 \le 110$$
	$$1000\le t3 \le 3000$$
	$$2\le t4 \le 20$$
Gear train problem	
Minimize:	${f}_{1}\left(t\right) = {(\left(\frac{1}{6.931}\right)-(\frac{t(1)t(2)}{t(3)t(4)}))}^{2}$
Minimize:	${f}_{2}\left(t\right) ={max}([t\left(1\right)t\left(2\right) t\left(3\right)t(4)])$
Where:	$12\le t\left(1\right), t\left(2\right), t\left(3\right), t(4)\le 60$
